# A Novel *Trypanosoma cruzi* Protein Associated to the Flagellar Pocket of Replicative Stages and Involved in Parasite Growth

**DOI:** 10.1371/journal.pone.0130099

**Published:** 2015-06-18

**Authors:** Ignacio M. Durante, María de los Milagros Cámara, Carlos A. Buscaglia

**Affiliations:** Instituto de Investigaciones Biotecnológicas-Instituto Tecnológico de Chascomús (IIB-INTECh), Universidad Nacional de San Martín (UNSAM) and Consejo Nacional de Investigaciones Científicas y Técnicas (CONICET); Buenos Aires, Argentina; University at Buffalo, UNITED STATES

## Abstract

The flagellar pocket constitutes an active and strategic site in the body of trypanosomatids (i.e. parasitic protozoa that cause important human and/or livestock diseases), which participates in several important processes such as cell polarity, morphogenesis and replication. Most importantly, the flagellar pocket is the unique site of surface protein export and nutrient uptake in trypanosomatids, and thus constitutes a key portal for the interaction with the host. In this work, we identified and characterized a novel *Trypanosoma cruzi* protein, termed TCLP 1, that accumulates at the flagellar pocket area of parasite replicative forms, as revealed by biochemical, immuno-cytochemistry and electron microscopy techniques. Different *in silico *analyses revealed that TCLP 1 is the founding member of a family of chimeric molecules restricted to trypanosomatids bearing, in addition to eukaryotic ubiquitin-like and protein-protein interacting domains, a motif displaying significant structural homology to bacterial multi-cargo chaperones involved in the secretion of virulence factors. Using the fidelity of an homologous expression system we confirmed TCLP 1 sub-cellular distribution and showed that TCLP 1-over-expressing parasites display impaired survival and accelerated progression to late stationary phase under starvation conditions. The reduced endocytic capacity of TCLP 1-over-expressors likely underlies (at least in part) this growth phenotype. TCLP 1 is involved in the uptake of extracellular macromolecules required for nutrition and hence in *T*. *cruzi* growth. Due to the bacterial origin, sub-cellular distribution and putative function(s), we propose TCLP 1 and related orthologs in trypanosomatids as appealing therapeutic targets for intervention against these health-threatening parasites.

## Introduction

*Trypanosoma cruzi* is the causative of Chagas Disease, a major health and economic issue in Latin America for which no vaccine or appropriate drugs for large-scale public-health interventions are yet available [1]. This protozoan parasite has a digeneic life-cycle, which alternates between an insect vector (*Reduviidae*, *Triatominae*) and a mammalian host [2]. Transitions between hosts as well as from intracellular to extracellular niches (and *vice versa*) are accompanied by extensive remodeling of parasite intracellular transport, primary metabolism, and gene expression profiling; and thus in the overall cellular proteome and architecture [3]. Indeed, *T*. *cruzi* consists of a pleomorphic population composed of several developmental forms that can be clearly recognized [2]. In particular, epimastigotes (spindle-shaped, with visible flagellum) constitute the replicative, non-infective forms found in the insect midgut. They attach to the cuticle of the rectal epithelium of the insect vector, and differentiate into non-dividing, infective metacyclic trypomastigotes, in a process known as metacyclogenesis [4]. Metacyclic trypomastigotes are deposited on the mammalian host along with the insect faeces during a blood meal, gain access to internal body fluids via a skin lesion or a mucosal surface and subsequently invade a wide variety of cells, in which they transform into amastigotes (rounded forms, with no visible flagellum). After a series of division rounds within the cytoplasm of infected cells, amastigotes differentiate into trypomastigotes (with evident flagella and undulating membrane), which represent the infective, non-replicative mammalian form [2].

As part of their evolutionary adaptation, trypanosomatids have developed highly specialized cellular organelles and anatomical structures [3]. One defining characteristic of trypanosomatids is the presence of a single flagellum required for motility, which emerges from the cell body through the flagellar pocket (FP) [5, 6]. This region does not merely represent a cavity, but instead constitutes an active and strategic site in the parasite body, which participates in diverse processes such as cell polarity, morphogenesis and parasite replication [5, 6]. Importantly, the FP constitutes the only surface in the parasite body lacking the sub-pellicular microtubule layer; a highly stable, cross-linked network of cytoskeletal elements which lies underneath the plasma membrane and maintains the parasite form [5, 6]. Since sub-pellicular microtubules are too closely spaced to allow transport vesicles to access the plasma membrane, molecular traffic interchange associated to endo- and exocytotic events are sterically restricted to the FP [5, 6].

The plasma membrane of trypanosomatids is dominated by glycosylphosphatidyl inositol (GPI)-anchored molecules [7], which are internalized via clathrin-dependent endocytic mechanisms. These mechanisms were found to be essential and stage-regulated in trypanosomes [8]. Endocytosis and turnover of type I trans-membrane proteins is also clathrin-dependent, but it seems to require ubiquitylation of target cytoplasmic lysine residues [9]. This statement, however, cannot be generalized since it has been so far only demonstrated for a small subset of *Trypanosoma brucei* molecules [10]. The critical role of the FP in both kinds of endocytosis as well as in exocytotic events has been best outlined in *T*. *brucei*, in which the FP must accommodate export of abundant GPI-anchored proteins such as variant surface glycoprotein (VSG) or procyclin while simultaneously mediate the uptake of extracellular macromolecules required for nutrition [5, 6]. By restricting type I trans-membrane receptors for host macromolecules, such as transferrin, to the FP, these relatively invariant surface proteins are protected from exposure to the host immune system. In the case of VSGs, although covering the bulk plasma membrane, flagellar beating influences trafficking through the FP and contributes to the removal of VSG-immunoglobulin protein complexes from the cell surface and consequent recycling of free VSGs [11]. In *T*. *cruzi*, endocytic activity was found to be tightly related to acquisition of nutrients and metacyclogenesis, the latter process induced by nutritional stress [12]. Overall, secretion, selective retention, uptake and/or recycling of surface molecules at the FP are essential and thus tightly regulated processes in trypanosomatids, both in terms of efficient nutrition but also for immune evasion [11].

In addition to the endosomal and recycling apparatus, the FP is also precisely positioned with respect to other organelles and compartments of the secretory system such as the endoplasmic reticulum (ER), Golgi complex, multivesicular body-like structure, sorting and recycling endosomes, and the contractile vacuole, a specialized organelle required for osmoregulation and trafficking of GPI-anchored virulence factors [13]. Importantly, the FP displays considerable organizational complexity, and several clear boundaries that demarcate distinct structural and likely functional sub-domains can be established [3, 5]. The most prominent sub-domains are i) the FP collar, which is found at the neck of the FP, where the flagellum exits the parasite body; ii) the collarette, which is the point where the flagellum enters the cellular body an presumably serves to anchor the membrane and the axoneme of the flagellum; and iii) the FP lumen, an invagination of the outer membrane which is filled with a carbohydrate-rich matrix of poorly defined composition. The 2 former sub-domains are associated with organized structures that are connected across the membrane and cytoskeleton [5, 6].

Few proteins functioning in the context of the FP have been functionally analyzed. Interestingly, most of them turned out to be essential, because their loss and/or miss-targeting led to morphological defects that fatally disrupted cellular functions [5, 6]. This, together with the fact that endocytic processes are deeply involved in virulence, differentiation, survival and drug delivery in these health-threatening parasites [14], point to FP-associated molecules as particularly valuable therapeutic targets. In the present work, we identified a novel *T*. *cruzi* molecule which accumulates in the FP area of replicative forms (i.e. epimastigotes and amastigotes). This molecule, termed TCLP 1 (Trypanosomatid CesT-like Protein 1), is conserved among trypanosomatids, and bears 3 homology inferred domains: an N-terminal Ubiquitin-Like Domain (UBL) [15], a C-terminal PSD95/Dlg1/zo-1 (PDZ) domain, which is involved in protein-protein interaction phenomena [16], and a domain with structural homology to CesT (Chaperone for *E*. *coli* secretion of Tir), which is a multi-cargo chaperone conserved in enteropathogenic bacteria that contributes to the secretion of virulence factors through type III secretion systems (TIIISS) [17, 18]. Using *in silico*, biochemical and microscopy-based approaches along with the fidelity of a homologous expression system, we characterized this molecule and demonstrated that TCLP 1-over-expressing parasites showed reduced endocytic capacity and impaired survival under starvation conditions.

## Methods

### Ethics statement

Animal immunization, bleeding and handling was carried out in strict accordance with the recommendations of the Institutional Committee on the Ethics and Proper Use of Experimental Animals (Comité Institucional para el Cuidado y Uso de Animales de Experimentación, CICUAE-UNSAM). The protocol was approved by the CICUAE UNSAM (Permit Number: 3–2013), and all efforts were made to minimize animal numbers (only 1 albino rabbit was utilized) and suffering.

### *In silico* predictions and phylogeny analyses

Identification of nuclear localization signals (NLS) was performed using the online predictors *cNLS Mapper* and *NLStradamus*. For the identification of nuclear export signals (NES) and signal peptides (SP), the online servers *NetNES* and *SignalP 4*.*0* were used, respectively. To study TCLP 1 orthologs and paralogs, BLAST analysis using *BLAST Explorer* was performed. Only the CEST domain of TCLP 1 was used as input to identify homologous protein sequences annotated in the NCBI non-redundant database. After manual curation of the output (i.e. exclusion of less informative or truncated sequences), an alignment was built with a subgroup of the resulting orthologs (full-length versions), and a phylogeny tree was constructed. Protein sequence alignments were performed with *MUSCLE v3*.*7* [19], and ambiguous regions (i.e. containing gaps and/or poorly aligned) were removed with *Gblocks v0*.*91b* [20] before reconstructing a phylogeny tree using the Maximum Likelihood method implemented in the *PhyML program v3*.*0* under default settings [21]. The final phylogram is the consensus tree of 100 bootstrap replicates and was graphically modified for presentation. ClustalW alignments of “CEST-like” proteins were performed under default settings using the *MegAlign* program (*Lasergene*).

### 3D-Modelling of the TCLP 1 CEST motif

Templates for the construction of 3-D homology inferred models were obtained with the template identification tool implemented in the SWISS-MODEL online server [22], and used one-by-one to generate structural models under default settings in automated mode. Once generated, models were inspected for resolution, coverage and quality using the “Structure Assessment Tool” implemented in the same server [22]. Structural alignments of the resulting final templates and the TCLP 1 CEST motif were performed using the online resource PROMALS3D and Jpred3 [23], and graphically modified for presentation. Superimposition of the generated 3D models and the templates was performed using the *Pymol* software. Model quality estimates are expressed using QMEAN values, calculated as described [24].

### Analysis of *TCLP 1* predicted ORF

To experimentally validate the CL Brener clone *TCLP 1* open reading frame (ORF) annotated in TriTrypDB (http://tritrypdb.org/tritrypdb/), 1 x 10^8^ *T*. *cruzi* CL Brener parasites (a mixture of epimastigote, amastigote and trypomastigote forms) were homogenized in 1 mL of TRIzol reagent (Thermo), further partitioned in chloroform and centrifuged at 12,000 *x g*. The aqueous phase containing the RNA was recovered and precipitated with 1 mL of 2-propanol. Total RNA was resuspended in RNAse-free H_2_O and used at a final concentration of 0.25 μg/μL and 3.5 μM oligo-dT primer (sequence and features of primers are indicated in S1 Table) in the reverse transcriptase (RT) First Strand Synthesis kit (Sigma). RT reactions were diluted and used as templates for PCR using oligonucleotides TcMe2, which anneals to the splice-leader sequence that is post-transcriptionally added to the 5' end of nearly all trypanosomatid immature polycistronic mRNAs [25], and TCLP 1 260 bp as reverse primer. PCR products were fractionated in 1.5% agarose gels, cloned into pGEM-T vector (Promega) and sequenced at our own facility.

### Real-time qPCR (quantitative PCR)

To evaluate *TCLP 1* mRNA expression, Real-time qPCR assays were conducted on cDNA samples prepared from epimastigote, amastigote and trypomastigote forms of *T*. *cruzi* CL Brener strain as described above. Real time qPCR assays were carried out using SYBR green-based Real-Time PCR system (Bio-Rad) as previously described [26] and results were normalized to ribosomal 18S mRNA expression [27]. Sequence and features of primers used are indicated in S1 Table.

### Cloning and expression of TCLP 1 in trypanosomes and bacteria

The most likely ORF of *TCLP 1* (TcCLB.510241.10), starting from the ATG codon placed 211 bp downstream of the initial ATG codon annotated in TriTryp DB, was PCR-amplified from *T*. *cruzi* (CL Brener strain) genomic DNA using TCLP 1 fwd and TCLP 1 rev primers, and cloned into pGEM-T vector [28]. To generate the TCLP 1::3xFLAG construct, *TCLP 1* was BamHI/NotI-digested and translationally fused to a 3xFLAG sequence cloned into the NotI/XhoI restriction sites of the pcDNA 3.1 vector (Invitrogen). EGFP::TCLP 1 was built by BamHI/XbaI sub-cloning of the TCLP 1::3xFLAG construct into pEGFP-C1 vector (Clontech), as a translational C-terminal fusion to EGFP. For *T*. *cruzi* homologous expression, TCLP 1::3xFLAG construct was BamHI/XhoI-digested from pcDNA 3.1 and cloned into pRIBOTEX [29]. Solely to assess the specificity of our αTCLP 1 antibody (see below), a truncated TCLP 1 version spanning the CEST domain and the TCLP 1 peptide (see below) was PCR-amplified from TCLP 1::3xFLAG construct using CEST pTrcHis fwd and rev primers, EcoRI-digested and cloned into pTrcHisC vector (Invitrogen). Cloning and expression procedures were performed in DH5α (Invitrogen) and BL21-Codon Plus (Stratagene) bacteria, respectively. All constructs were verified by sequencing at our own facility.

### Parasite growth and transfection

*T*. *cruzi* epimastigote forms from the CL Brener clone or the Adriana strain [26] were grown at 28°C in brain-heart tryptose (BHT) medium supplemented with 10% Fetal Calf Serum (FCS, Gibco)(BHT 10%). Epimastigotes from the Adriana strain (1.5 x 10^8^) were electroporated with 10 μg of purified DNA, and selected with 250 μg/mL of G418 (Invitrogen) [30, 31]. Parasites were not cloned by limited dilution or enriched by any means, and antibiotic selection was sustained over time once stably transfected populations were obtained. Growth curves of Adriana epimastigotes transfected with i) TCLP 1::3xFLAG (TCLP 1), ii) a 3xFLAG-tagged version of the complete ORF of the TcCLB.511277.250 gene (TI) or iii) a 3xFLAG-tagged version of the complete ORF of the TcCLB.510181.60 gene (TII) were performed in BHT 10% supplemented with G418 [26], unless otherwise stated. Conditioned medium (CM) was prepared as follows: late stationary phase parasites were centrifuged 5 min at 4,000 *x g* and the supernatant was removed and sterilized by filtration through a 0.22 nylon membrane (Corning). Cumulative growth rate (CGR) was calculated as: CGR (%) = 100 –(Number of parasites at 48 h/Number of parasites at 24 h x 100). Paired contrasts were performed with two-tailed Student’s T-tests implemented in the *GraphPad Prism* software. When required, *T*. *cruzi* cell-derived trypomastigote and amastigote forms from the CL Brener clone or the Adriana strain were obtained and purified as described [32].

### Antibody production

Keyhole limpet hemocyanine-coupled TCLP 1 peptide (^637^TSPSKNGHSTIEDC-klh) was purchased from GenScript and used to inoculate an albino rabbit according to standard procedures [33]. Serum aliquots were analysed using TCLP 1 peptide-coated ELISA plates as described [34, 35]. Antibodies were affinity-purified from rabbit serum using the TCLP 1 peptide coupled via the C-terminal Cysteine residue to sulfo-link resin (Pierce) [26]. The sensitivity and specificity of the final antibody (referred to as αTCLP 1 throughout this work) were evaluated by means of Western blot and indirect immunofluorescence (IIF) assays. Displacement assays were performed by pre-incubating αTCLP 1 with Phosphate Buffer Saline (PBS) or the indicated peptide for 1 h at room temperature. The sequence of the mock peptide used for these assays was: EVDDSEEKEEIIQC.

### Manipulation and transfection assays in mammalian cell lines

HeLA cells were grown at 37°C, 5% CO_2_ in Dulbecco’s Modified Eagles Medium (D-MEM) supplemented with 10% FCS and antibiotics. Transient transfection was performed using Lipofectamine 2000 reagent (Invitrogen) as recommended by the manufacturer. For epi-fluorescence microscopy, cells were washed in PBS, fixed in PBS 4% *p*-formaldehyde (PBS-PAF) for 10 min and processed for IIF assays (see below).

### Parasite fractionation, secretion extracts, Western blot and flow cytometry analyses

Nonidet P40-based fractionation was performed as described elsewhere [30]. The resulting fractions (Pellet, Cytoplasm and Microsomes) were run in 12.5% SDS-PAGE, transferred to PVDF membranes (GE Healthcare), and probed with mAb anti-FLAG (clone M2, Sigma) followed by HRP-conjugated anti-mouse IgG (Sigma) (both at 1:5,000 dilution) and the chemoluminescence substrate West-Fempto ECL reagent (Pierce). Anti-TcPaBP [36] and anti-TbBiP [37] were used as controls of cytoplasmic and microsomal fractions at 1:3,000 and 1:1,000 dilution, respectively [30]. Secretion extracts were obtained from 2 x 10^8^ parasites as described elsewhere [30], and the resulting fractions (Pellets and Secretion Extracts) were processed for Western blot. Two recombinant parasites stably transfected with FLAG-tagged versions of either full-length (surface associated and secreted) or truncated TcSMUG S product (TcSMUG SΔΔ, cytoplasmic and non-secreted) were used as controls for these experiments [30]. TCLP 1 expression on saponin-permeabilized epimastigotes was evaluated by staining with αTCLP 1 at 1:10 dilution followed by flow cytometry analysis [30].

### Extracellular and intracellular parasite epi-fluorescence microscopy

For IIF assays, parasites were harvested, washed in PBS, adhered to poly-L-lysine (Sigma) coated cover-slips, fixed for 30 min in 4% PAF-PBS, blocked for 30 min in 4% Bovine Serum Albumin (Sigma) in PBS (PBS-A) supplemented with 0.5% saponin (Sigma) for permeabilization, and probed with the indicated antibody diluted in PBS-A [26, 31]. After extensive washings with PBS, secondary Alexa Fluor-conjugated antibodies (Molecular Probes) were added. Nuclei were stained with DAPI prior to montage in FluorSave reagent (CalBiochem). Polyclonal anti-TbGRASP [38] and anti-TcCruzipain [39] antibodies were used at 1:500 dilution, polyclonal anti-TcAquaporin antibody [13] was used at 1:200 dilution, and mAbs anti-FLAG and FK-2, that recognizes mono-and poly-ubiquitylated proteins (Merck-Millipore) were used at 1:1,000 dilution. To evaluate the reactivity of *T*. *cruzi* intracellular stages 10,000 Vero cells were plated onto round coverslips, let stand overnight and infected with 1 × 10^6^ CL Brener trypomastigotes per coverslip. Following 96 h, the infected cells were washed with PBS, fixed and processed as described [32]. Images were obtained with a Nikon Eclipse 80i epi-fluorescence microscope coupled to a DS-Qi1 CCD camera, and processed using *ImageJ*.

### Endocytosis assays

Concanavalin A (ConA) and transferrin internalization assays were performed as described elsewhere [40]. Briefly, parasites were washed twice in PBS and incubated 10 min on ice in FCS-free BHT medium supplemented with 100 μg/mL of either ConA-rhodamine or Transferrin-Alexa 488 conjugate (both from Invitrogen). Cells were then placed at 28°C to allow for dye uptake and aliquots were taken at 0 seconds, 15 seconds and 5 min, placed on ice, fixated with 4% PAF-PBS and processed for epifluorescence analysis as above. For uptake quantitation, background corrected images of TCLP 1 and parental, wild type parasites were used; and fluorescence intensity at polygonal regions corresponding to the FP area was measured using *ImageJ*. For each strain/time point, a minimum number of 100 parasites were evaluated. Mean intensity values were obtained and the statistical significance of results was evaluated by paired contrasts with two-tailed Student’s T-tests implemented in the *GraphPad Prism* software. For flow cytometry-based quantitation, parasites were washed twice in PBS and incubated for 10 min on ice in FCS-free BHT medium supplemented with 100 μg/mL of either ConA-rhodamine or Transferrin-Alexa 488 conjugate. Cells were then placed at 28°C to allow for dye uptake and aliquots were taken at 0, 2 and 15 min, placed on ice, fixated with 4% PAF-PBS and processed using fluorescence-activated cell sorting CyFLOW Partec and FloMax software as described [30].

### Transmission electron microscopy and immunoelectron microscopy

For transmission electron microscopy (TEM) analysis, 1 x 10^9^ parasites were washed 3 times with 0.2 M phosphate buffer pH 7.5 (PB), fixed on ice for 1 h with 0.25% glutaraldehyde, 4% PAF-PB, and washed 3 times with PB. Samples were embedded in LR-White resin, sectioned and prepared for TEM according to standard procedures of the LANAIS-MIE (UBA-CONICET) electron microscopy facility. For immunoelectron microscopy, samples were stained with either mAb anti-FLAG or αTCLP 1 at 1:100 and 1:10 dilution, respectively, followed by 5- or 10-nm gold-conjugated secondary antibodies (Sigma). Ultra-thin sections were observed under a Zeiss EM 109-T and images were obtained with a coupled Gatan ES1000W CCD camera. Image processing was performed with *ImageJ*.

## Results

### TCLP 1 belongs to a novel, chimeric family of proteins restricted to trypanosomatids

TCLP 1 emerged as a top-ranked candidate during an *in silico* screening aimed at identifying proteins potentially involved in modulating host cellular processes during a *T*. *cruzi* infection (to be published elsewhere). When setting this screening, we took into account several biologically-relevant criteria such as expression data, presence of protein secretion features (i.e., N-terminal signal peptide (SP), consensus GPI-attachment signal [30] and potential to be secreted via a leaderless secretion pathway, including exosome-like vesicles [41]), presence of a "nuclear features" (i.e., nuclear trafficking and/or nucleic acid-binding domains); presence of UBL or UBL-binding domains among others [42]. To counterbalance possible errors in the assembly/annotation of the *T*. *cruzi* genome [43], and/or biases in the training of the different algorithms, we avoided selecting proteins by asking them to strictly meet all required criteria, as in a regular screening. Instead, we opted for a more inclusive strategy using scores, in which every protein was assigned a numeric value (positive or negative) for each finding. This 'ranked' output strategy has been employed in the prioritization of targets for drug discovery [44].

**Fig 1:**
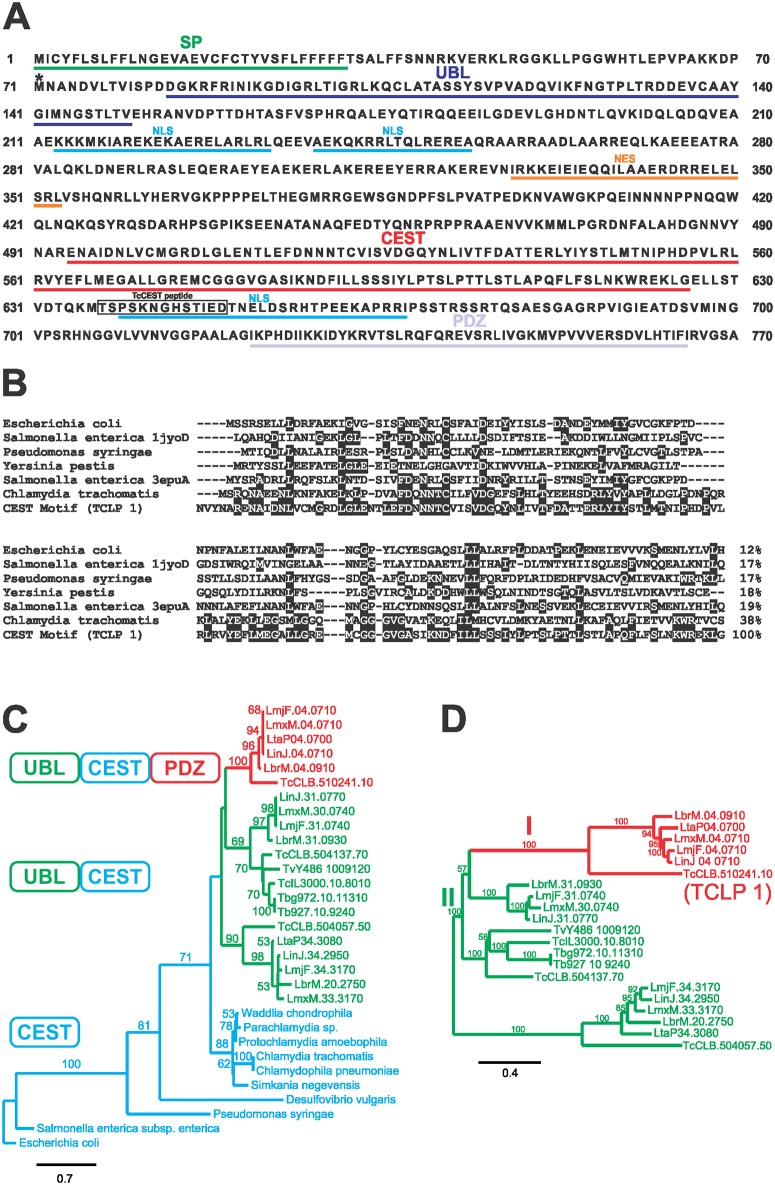
TCLP 1 belongs to a novel, multi-domain family of proteins conserved in trypanosomatids. (A) *In silico* predictions and annotated motifs are underlined in the TCLP 1 deduced sequence from the CL Brener clone of *T*. *cruzi*. Asterisk denotes the actual initial Methionine residue identified in this work for TCLP 1. Abbreviations: SP, Signal Peptide; UBL, Ubiquitin-Like domain; CEST, CesT-like domain; PDZ, PSD95/Dlg1/zo-1 domain; NLS, Nuclear Localization Signal; NES, Nuclear Export Signal. The cognate peptide recognized by the αTCLP 1 antibody is boxed. (B) A simplified Clustal-W alignment of TCLP 1 CEST motif and most relevant bacterial CEST molecules. Conserved residues are shaded, and overall amino acid identity with TCLP 1 (in %) is indicated to the right. (C) A phylogeny tree of "CEST-like" molecules identified in trypanosomatids. The tree was rooted with the lowest branch as outgroup (*Escherichia coli*). Trypanosomatid “CEST-like” proteins are named with their corresponding Gene ID. The domain architecture of Class I (UBL-CEST-PDZ) and Class II (UBL-CEST) trypanosomatid “CEST-like” proteins and bacterial CEST molecules is indicated by a color code. (D) 100 bootstrap tree derived of a Clustal-W alignment of selected trypanosomatid "CEST-like" proteins. Bootstrap support values (in %) for the main branches are indicated. The scale indicates the amino acid substitution distance along the branches. Abbreviations: TcCLB, *T*. *cruzi* CL Brener clone; MOQ, *T*. *cruzi marinkellei*; TCSYLVIO, *T*. *cruzi* Sylvio X-10 strain; Lbr, *Leishmania braziliensis;* Lta, *L*. *tarentolae;* Lmx, *L*. *mexicana;* Lmj, *L*. *major;* Lin, *L*. *infantum;* LDBPK, *L*. *donovani;* Tb, *T*. *brucei;* Tbg, *T*. *brucei gambiense;* TcIL, *Trypanosoma congolense;* TvY, *Trypanosoma vivax*.

The deduced protein from the *TCLP 1* gene of the CL Brener clone (TcCLB.510241.10) is annotated in TriTrypDB as a conserved hypothetical protein bearing a putative SP and 3 homology inferred domains: i) an N-terminal UBL motif similar to that found in Rad23 [45]; ii) a domain (IPRO10261) with homology to the chaperone CesT (henceforth the CEST domain) involved in the secretion of virulence factors through TIIISS in enteropathogenic bacteria; and iii) a putative PDZ domain towards the C-terminus (Fig 1A). Further *in silico* analyses allowed us to identify two putative NLS on TCLP 1: one located between the CEST and the PDZ domains, and a bi-partite motif between the UBL and the CEST domains (Fig 1A). In addition, a “weak”, i.e. only recognized by the Hidden Markov Model of the predictor, NES located between the UBL and the CEST domains was also identified (Fig 1A).

The finding of a prokaryotic domain in TCLP 1 prompted us to conduct a series of studies aimed at characterizing this protein in further detail. A PSI-BLAST-based analysis showed an overall low sequence identity (12–38%) between the TCLP 1-derived CEST motif and bacterial CesT molecules (Fig 1B). Multiple pair-wise comparisons, however, revealed low sequence similarities even when comparing between bacterial CesT sequences (not shown), suggesting that low sequence conservation is a common issue between TIIISS chaperones. TCLP 1 CEST motif most significant similarities (~38%) were found with molecules from the *Chlamydiae/Verrucomicrobia* taxa (Fig 1B), a group of obligate intracellular pathogens that express a functional TIIISS during their infection cycle [46]. Additional BLAST analyses using the CEST domain of TCLP 1 allowed for the identification of several hypothetical, CEST-containing proteins in other *T*. *cruzi* isolates as well as in different *Trypanosoma* and *Leishmania* species (S1 Fig). At variance with bacterial molecules in which the CEST motif spans almost their entire ORFs and constitutes their unique functional domain [17, 18], trypanosomatid "CEST-like" proteins are chimeric molecules with rather conserved architecture (Fig 1C and S1 Fig). More precisely, all of them have an additional N-terminal UBL domain, and in some cases they also bear a C-terminal PDZ motif (Fig 1C and S1 Fig). According to their domain composition, trypanosomatid “CEST-like” proteins were split into two distinct groups: Class I, formed by UBL-CEST-PDZ containing proteins and Class II, composed of UBL-CEST containing but PDZ lacking proteins (Fig 1C). The Class I group, which is the most divergent monophyletic group, comprises the TCLP 1 product deduced upon the CL Brener *TCLP 1* gene (TcCLB.510241.10), the product deduced upon the allele variant found in the Sylvio X-10 strain of *T*. *cruzi* (TCSYLVIO_007169,) and its putative orthologs found in the bat parasite *T*. *cruzi marinkellei* (MOQ_005905) and in different *Leishmania* spp (Fig 1D and not shown). Several Class II molecules can be found in different strains of *T*. *cruzi*, *T*. *cruzi marinkellei*, *Leishmania* and also African trypanosomes (Fig 1D). According to these findings, and to the fact that neither Class I nor Class II "CEST-like" proteins were found outside of the trypanosomatid branch of eukaryotes, the most parsimonious hypothesis would imply that i) Class I “CEST-like” proteins originated upon an early horizontal gene transfer (HGT) of the CEST domain from the *Chlamydiaceae* into some trypanosomatid ancestor; ii) Class II members aroused due to Class I gene duplication and subsequent loss of the PDZ domain; and iii) Class I proteins would have then been secondarily lost in the evolutionary branch of African trypanosomes.

### TCLP 1 displays structural similarity to *Salmonella* TIIISS chaperones

**Fig 2:**
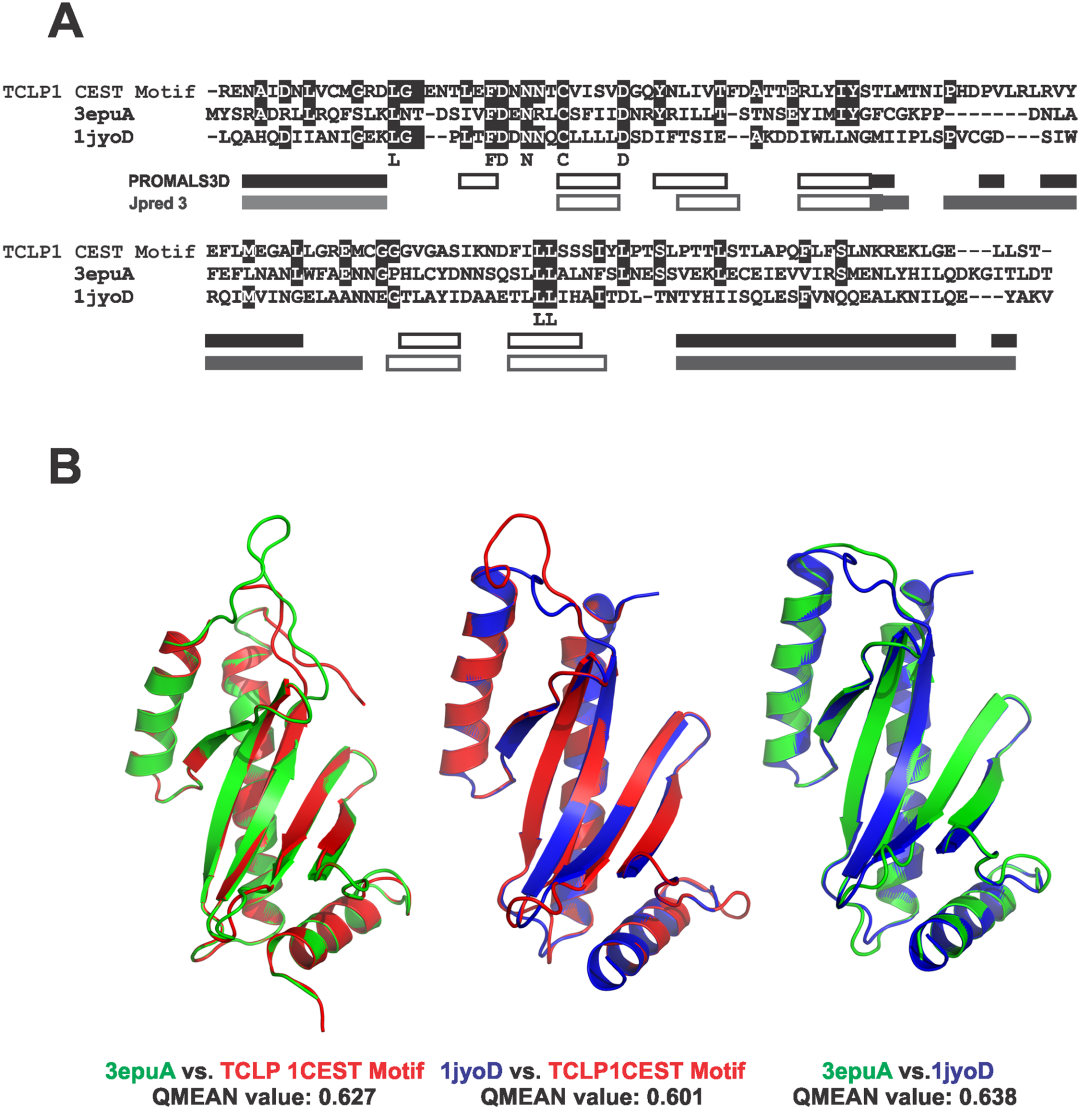
Structural conservation between TCLP 1 and *Salmonella* TIIISS chaperones: (A) The sequences of the TCLP 1-derived CEST motif, 3epuA and 1jyoD were aligned with the program PROMALSD3D. Secondary structure motifs predicted for the consensus sequence obtained for both bacterial molecules are depicted below as solid (α helices) or empty (ß sheets) black boxes. Secondary structure motifs independently predicted for the TCLP 1 CEST motif using Jpred3 analysis are shown below as solid (α helices) or empty (ß sheets) grey boxes. (B) Model superimposition of the TCLP 1 CEST motif with templates 3epuA and 1jyoD. *Left*, CEST motif model I (3epuA-based, red) and 3epuA structure (green). *Middle*, CEST motif model II (1jyoD-based, red) superimposed with 1jyoD structure (blue). *Right*, Model of 1jyoD, 3epuA-based (green), superimposed with 1jyoD structure (blue). QMEAN values are shown below each panel.

To further characterize the TCLP 1-derived CEST domain, a search for suitable templates was conducted using the online SWISS-MODEL Workspace [22]. In the absence of chlamydial templates, 2 *Salmonella enterica*-related templates, namely 3epuA and 1jyoD, were selected to perform structural alignments. The former PDB structure corresponds to STM2138 TIIISS chaperone X-ray diffraction structure, with a 2.5 Å resolution [47] whereas the latter corresponds to the TIIISS homo-dimeric chaperone SicP X-ray diffraction structure, solved at 1.9 Å resolution [48]. Despite showing poor sequence conservation with the TCLP 1 CEST motif (19% and 17% identity either for 3epuA or 1jyoD, respectively, Fig 2A), the assignment of secondary structural motifs (α-helices and ß-sheets) for the consensus of both molecules by means of the PROMALS3D algorithm nicely superimposed with that obtained independently for the TCLP 1-derived CEST motif using the Jpred3 predictor (Fig 2A). Significantly, the resulting structural models of the TCLP 1 CEST motif constructed upon these templates showed reasonable superimposition of the buried, hydrophobic ß-sheets and the outer α-helices previously described for the SicP chaperone [48] (Fig 2B). The QMEAN values for these 2 models were 0.627 and 0.601, respectively, which can be considered acceptable [24]. Moreover, when the sequence of 3epuA was superimposed to the 1jyoD structure, the resulting QMEAN value was 0.638, thus quite similar from those obtained for the TCLP 1-derived CEST motif (Fig 2B). Taken together, these results suggest that despite its low sequence conservation, the CEST motif present in TCLP 1 displays significant structural similarity with distant TIIISS chaperones from bacterial origin.

### TCLP 1 is majorly expressed as a SP-less product in replicative stages of *T*. *cruzi*

As mentioned TcCLB.510241.10 is annotated as an hypothetical 776 amino acids-long protein bearing a predicted N-terminal SP (Fig 1A). However, this putative SP was not found in TCSYLVIO_007169 or MOQ_005905; neither in any of the remainder TCLP 1 orthologs/paralogs identified in trypanosomatids (S1 Fig). This fact suggested that the SP prediction may be an artifact resulting from an erroneous annotation, which is not an uncommon issue in trypanosomatids [43, 49]. Further supporting this possibility, an *in silico* analysis by means of a program specifically designed for *T*. *cruzi* ORF prediction (Serra et al., unpublished results), showed that the “P-values for coding” for TcCLB.510241.10 shift from 0 to 1 at 211 bp downstream of the predicted start-codon (S2 Fig). It is worth noting that TcCLB.510241.10 has an additional in-frame ATG at this position (S2 Fig), which if indeed used for translation initiation, would yield a shorter-than-predicted and, more importantly, SP-less TCLP 1 product. This additional in-frame ATG is annotated as the actual start codon for TCSYLVIO_007169 and MOQ_005905 (S1 Fig). Given the structural and functional implications of the presence/absence of an N-terminal SP, we undertook a well-established molecular approach to address this issue [49]. To that end, samples of total CL Brener RNA from different developmental forms were used to perform RT-PCR with a forward primer directed against the *T*. *cruzi* splice-leader and a reverse primer specific to a region ~260 bp downstream of the TcCLB.510241.10-predicted start-codon (S2 Fig). A unique product of ~170 bp was obtained when the reaction was performed in the presence of RT but was absent in mock controls in which the enzyme was omitted (S2 Fig). The RT-PCR product was cloned and several independent colonies (*n* = 15) were sequenced. As shown in S2 Fig, a unique *trans*-splicing acceptor site for TcCLB.510241.10 was consistently found, which is placed at 115 bp downstream of its predicted start codon. The first in-frame AUG of the identified mRNA species is the one located 211 bp downstream of the TcCLB.510241.10-predicted start-codon, which is the start codon identified by the *T*. *cruzi* ORF predictor (see above) and the initial ATG annotated for TCSYLVIO_007169 and MOQ_005905 (S1 and S2 Figs). Identical results were obtained when RT-PCR analyses were carried out in epimastigotes from the Adriana strain (not shown). Although these results do not completely rule out the existence of putative TcCLB.510241.10 mRNAs using different *trans*-splicing acceptor sites in parasite forms not analyzed here (such as metacyclic trypomastigotes) or grown under different conditions, they allowed us to conclude that *TCLP 1* exists as a major, if not unique, transcriptional form in *T*. *cruzi* developmental stages analyzed in this work, which codes for a SP-less TCLP 1 protein product.

**Fig 3:**
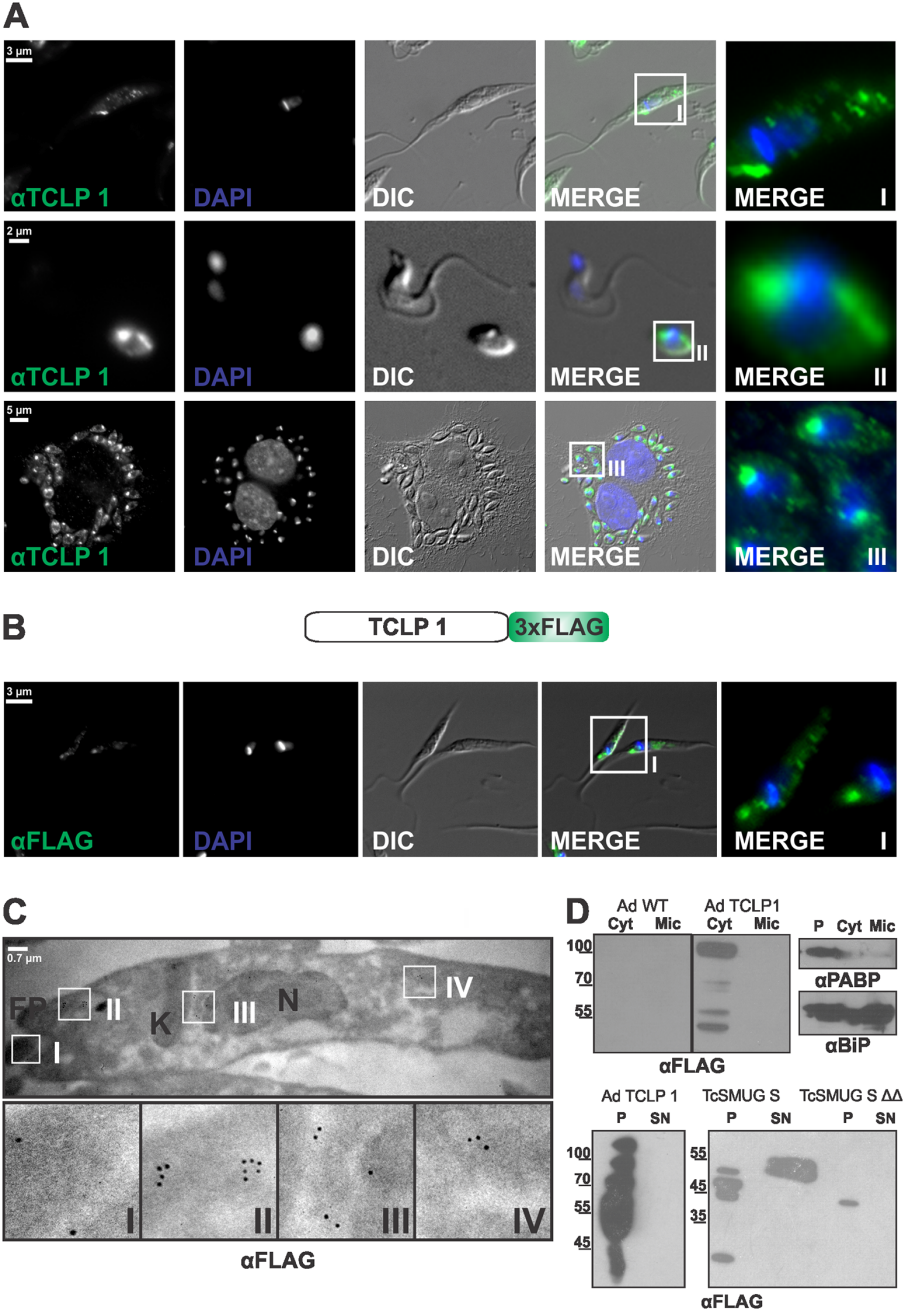
TCLP 1 is expressed in *T*. *cruzi* replicative forms. (A) Epimastigotes (upper panels), trypomastigote/amastigote mixtures (middle panels) and HeLa cells infected with amastigotes (all from CL Brener strain) were permeabilized and analyzed by IIF using anti-TCLP 1 antibody (αTCLP 1, green). DAPI signals are shown in blue. Enlarged regions (I, II and III) are depicted with rectangles in the original merge images. (B) Adriana Epimastigotes transfected with TCLP 1::3XFLAG (Ad TCLP 1) were analyzed by IIF using the monoclonal anti-FLAG antibody (αFLAG, green). DAPI signals are shown in blue. An enlarged area depicting the flagellar pocket (FP) region is shown with a rectangle in the original merge image. (C) Immunoelectron microscopy of Ad TCLP 1 epimastigotes labeled with αFLAG (10-nm gold particles). A longitudinal section of the parasite highlighting the FP, Nucleus (N) and Kinetoplast (K). Enlarged areas showing αFLAG reactivity in the FP (I), N/K (II) and the posterior region of the epimastigote are indicated with rectangle in the original image. (D) Upper left panel, Cytoplasm (Cyt) and Microsomal (Mic) fractions from wild type Adriana (Ad WT) and Ad TCLP 1 parasites were probed with αFLAG. Upper right panels, similar fractions (including parasite pellets, P) from Ad TCLP 1 parasites were probed with anti-PABP (cytoplasmic marker) or anti-BIP (microsomal marker) antibody. Lower panels, Total lysates (P) and secretion extracts (SN) from Ad TCLP 1 or from Adriana epimastigotes transfected with FLAG-tagged constructs of TcSMUG S or TcSMUG S ΔΔ (see Materials and Methods) were probed with αFLAG. Molecular mass markers (in kDa) are indicated to the left.

Expression of the TCLP 1 product was determined by IIF assays probed with an affinity-purified antibody (αTCLP 1) directed against a unique (i.e. not found in other *T*. *cruzi* "CEST-like" proteins) TCLP 1 peptide (Fig 1A and S1 Fig). The specificity of αTCLP 1 was assessed by Western blot and IIF assays using TCLP 1 products expressed in different heterologous and homologous systems (S3 Fig). As shown in Fig 3A, TCLP 1 is present in parasite replicative forms (i.e. epimastigotes and amastigotes) but not in trypomastigotes, which is in close agreement with previous transcriptomic data ([50] and S2 Table). Quantitative Real-Time PCR assays further supported this expression profile for *TCLP 1* (S2 Fig). Notably, in both replicative stages TCLP 1 accumulates at the base of the flagellum in an anterior position with respect to the kinetoplast, thus coincident with the FP area (Fig 3A). In some cases, αTCLP 1 also yielded a less intense and punctuated labeling pattern in the posterior part of both parasite forms (Fig 3A). The αTCLP 1 signal in parasites was abolished when the antibody was pre-adsorbed with the cognate TCLP 1 peptide (S3 Fig). Unfortunately, the αTCLP 1 antibody did not clearly label any *T*. *cruzi* developmental stage in Western blotting over total parasite lysates or secretion extracts (S3 Fig and not shown), thus precluding its use for further biochemical analyses. To overcome this hurdle, we decided to exploit the fidelity of an homologous expression strategy. To that end, a 3xFLAG-tagged version of TCLP 1 was generated in the *T*. *cruzi* expression vector pRIBOTEX [29]. As mentioned above, and based on our previous *in silico* and experimental findings (S1 and S2 Figs), this construct (termed TCLP 1::3xFLAG) started from ATG_211_, and thus did not include the N-terminal SP annotated for TcCLB.510241.10. Upon transfection of this construct in *T*. *cruzi* epimastigote forms, TCLP 1 expression was significantly increased as determined by Western blot and flow cytometry (S3 and S4 Figs). The TCLP 1::3xFLAG product showed almost indistinguishable sub-cellular distribution than the endogenous TCLP 1 product (compare Fig 3A and 3B). As observed in wild type parasites (Fig 3A), some TCLP 1::3xFLAG-transfected epimastigotes (henceforth, TCLP 1 epimastigotes) showed, in addition to the signal accumulation in the FP area, some discrete accumulations in the posterior part of the parasite body (Fig 3B). The specificity of the FLAG signal in our IIF assays is assessed in S3 Fig. TEM images of TCLP 1 epimastigotes labeled with mAb anti-FLAG further supported this sub-cellular distribution. As shown in Fig 3C, gold particles accumulated in the intracellular FP area and, to a lesser extent, in the vicinity of the kinetoplast, in the perinuclear area and in the posterior part of the parasite body.

**Fig 4:**
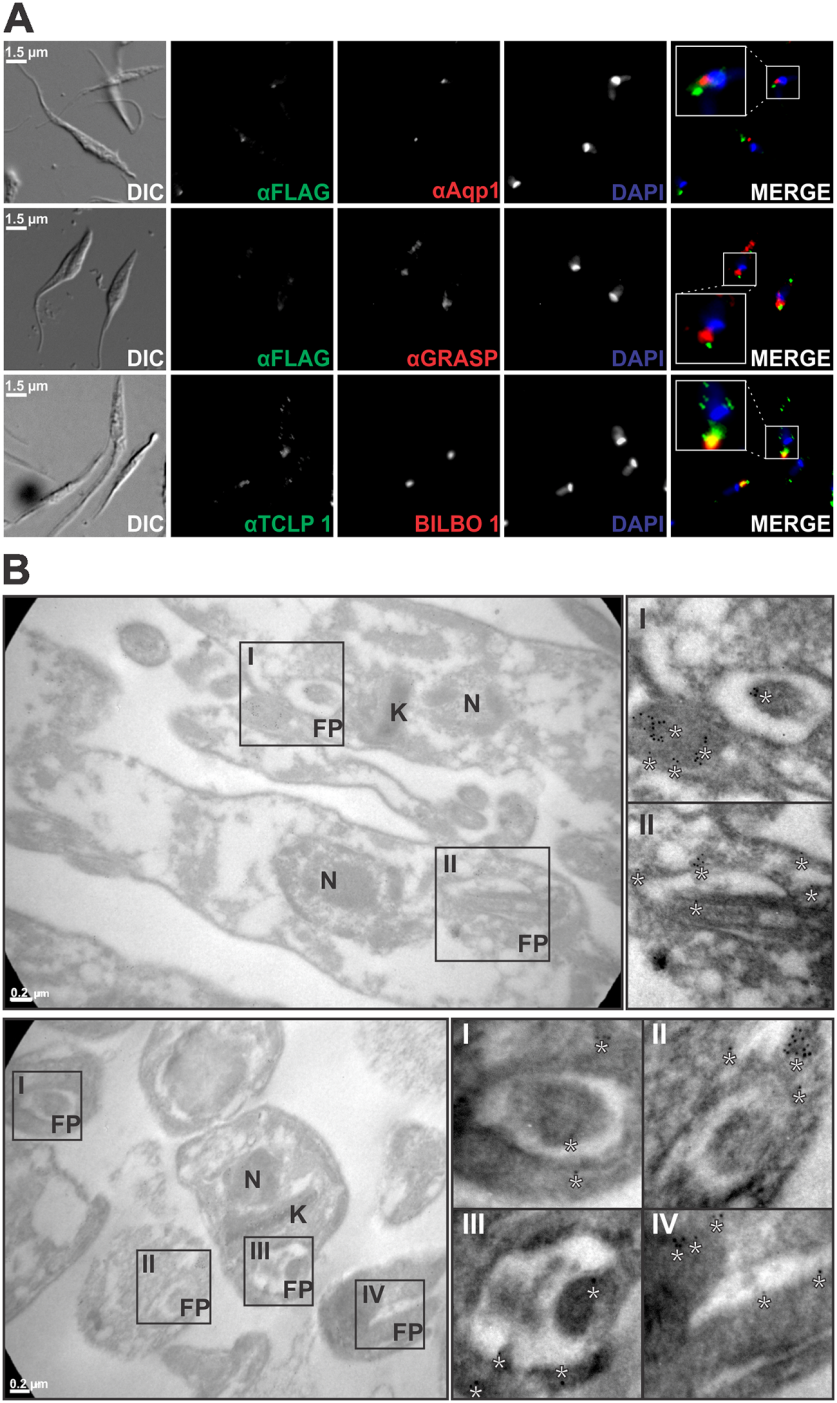
Fine sub-cellular localization analysis of TCLP 1 in *T*. *cruzi*. (A) IIF assays of TCLP 1::3XFLAG-transfected epimastigotes were probed with monoclonal anti-FLAG antibody (αFLAG, green) and co-stained either with anti-Aquaporin 1 (αAqp, upper panels) or anti-GRASP (αGRASP, middle panels) antibody (both in red). Lower panels, TcBilbo-1::mCherry-transfected parasites (BILBO, red) were stained with anti-TCLP 1 (αTCLP 1) antibody (green). DAPI signals are shown in blue and enlarged rectangular regions are shown in merged images. (B) Immunoelectron microscopy of wild type CL Brener epimastigotes (upper panels) and amastigotes (lower panels) labeled with αTCLP 1 (5-nm gold particles). Longitudinal sections of the parasite forms highlighting the flagellar pocket (FP), Nucleus (N) and Kinetoplast (K). Enlarged areas showing αTCLP 1 reactivity in the FP are indicated with rectangles in the original images. Asterisks highlight gold particles. Bar scale, 0.2 μm.

Western blot analyses allowed for the detection of major FLAG-reactive bands of ~100, ~75, ~50 and <25 kDa in total lysates from TCLP 1 epimastigotes (Fig 4D and S3 Fig). Given that the predicted molecular mass of the TCLP 1::3xFLAG product is ~75 kDa, FLAG-tagged products showing higher- and lower-than-expected molecular masses could be in principle attributed to post-translational modifications and/or degradation phenomena. Indeed, the αTCLP 1 antibody was unable to recognize the higher molecular weight bands in TCLP 1 epimastigotes, suggesting possible steric hindrance and/or epitope blockage effects due to post-translational modifications events (S3 Fig). Sub-cellular fractionation assays indicated that the TCLP 1::3xFLAG product is present exclusively in the cytoplasmic fraction (Fig 3D). Even though this reporter does not include the TcCLB.510241.10-predicted SP, Western blot analysis of secretion extracts from transgenic parasites was yet considered as relevant, given the reported significance of “non-canonical” secretion pathways in trypanosomatids [41]. Moreover, 2 of *Leishmania infantum* “CEST-like” proteins (lacking predicted SP, S1 Fig) were indeed found in the *Leishmania* secretome ([51] and S2 Table). Despite these considerations, the TCLP 1::3xFLAG product was not detected in secretion extracts of transgenic epimastigotes, whereas a recombinant, FLAG-tagged mucin that is actively secreted to the milieu [30] was indeed detected (Fig 3D). Together, these results indicate that TCLP 1 accumulates in cytoplasmic locations associated to the FP area of parasite replicative forms.

### TCLP 1 co-localizes with molecular and functional markers of the FP area

To more accurately circumscribe TCLP 1 sub-cellular distribution, a series of co-localization experiments were conducted. Molecular markers of the contractile vacuole (Aquaporin-1, [13]) and the Golgi apparatus (GRASP, [38]) did not co-localize with TCLP 1::3xFLAG or endogenous TCLP 1 (Fig 4A and not shown). As expected, both markers were found to be in close proximity to the FP area (and thus to TCLP 1), although at a slightly posterior localization. Bilbo-1, a cytoskeletal protein originally identified and characterized in *T*. *brucei*, is so far the only molecular marker of the FP collar [52]. Recently, this protein was cloned and recombinantly expressed in *T*. *cruzi* epimastigotes, where it also localized to the FP [53]. IIF assays of pTREXL::mCherry::Bilbo-1-transfected parasites indeed showed co-localization between TCLP 1 and Bilbo-1 signals (Fig 4A). As a complementary analysis, TEM images from ultra-thin sections of CL Brener wild type epimastigote and amastigote forms were probed with the αTCLP 1 antibody to determine its localization at the ultra-structural level. As shown, gold particles accumulated in close proximity to the FP area in both parasite developmental forms (Fig 4B). It is worth noting, however, that TCLP 1 was not detected in the FP membrane and/or lumen, but exclusively at intracellular and likely cytoplasmic locations. More precisely, TCLP 1 was detected either underneath the FP membrane, in the FP collar/collarette or along the body of the emerging flagellum (Fig 4B and not shown). As shown for the TCLP 1::3xFLAG product (Fig 3C), gold particles also accumulated though to a lesser extent, in the vicinity of the kinetoplast, in the perinuclear area and in the posterior part of the parasite body (Fig 4B).

**Fig 5:**
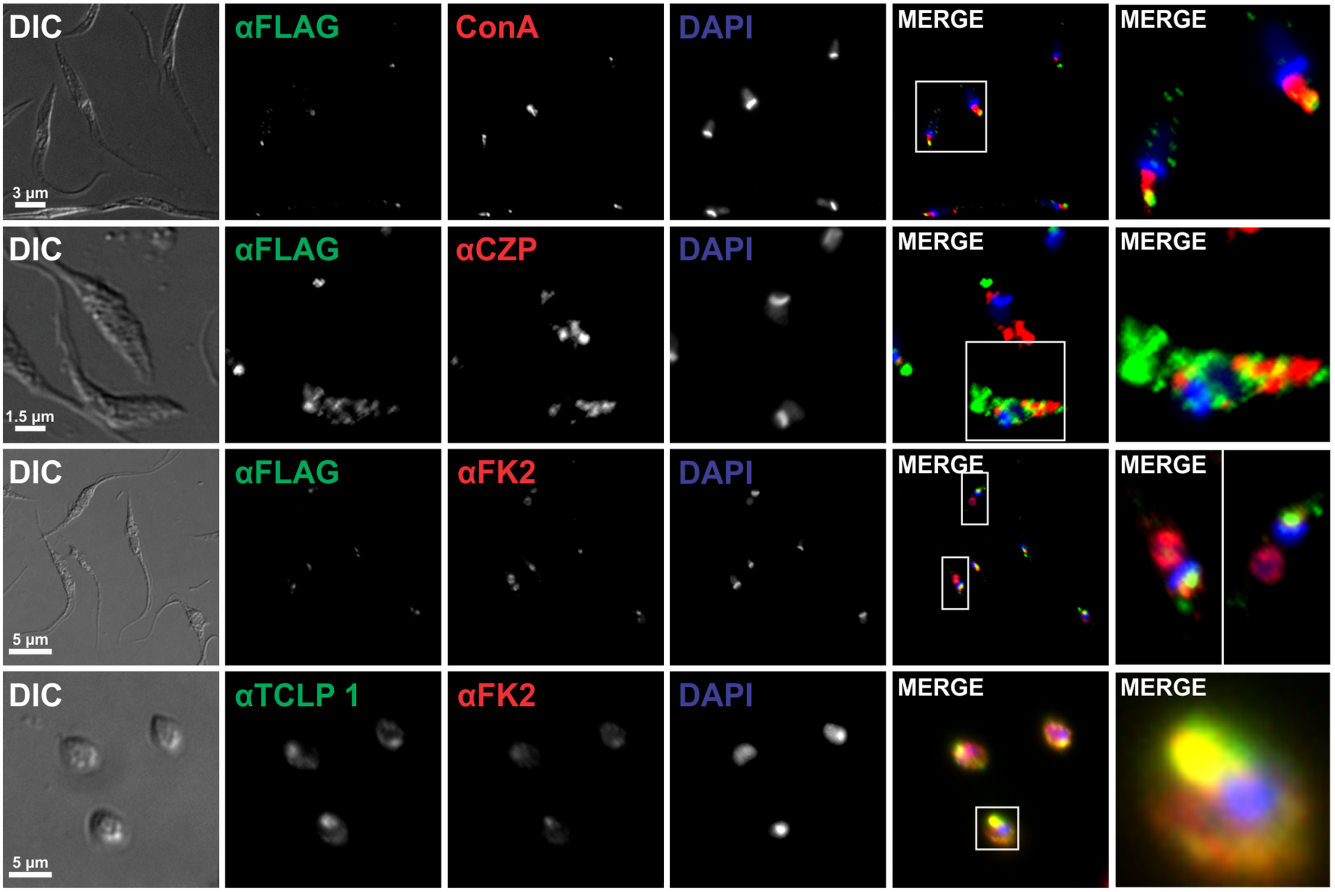
TCLP 1 co-localizes with markers of the endocytic but not degradative pathway. IIF assays of TCLP 1::3XFLAG-transfected epimastigotes or wild type Adriana amastigotes (lower panels) were probed with monoclonal anti-FLAG (αFLAG) or anti-TCLP 1 (αTCLP 1) antibody (green) and co-stained either with ConA-rhodamine (ConA), anti-cruzipain (αCZP) or monoclonal FK-2 (αFK2) antibody (all in red). DAPI signals are shown in blue and enlarged rectangular regions are shown in merged images.

As mentioned, exo- and endocytic processes are restricted to the FP in trypanosomatids [5]. Therefore, we performed a series of co-localization assays using functional markers of these processes. Upon incubation with rhodamine-labeled ConA, the overall endocytic system of *T*. *cruzi* epimastigotes became labeled, including the FP and posterior tubular compartments. As shown, TCLP 1 co-localized with ConA at initial (FP-associated) compartments, but not at posterior tubules (Fig 5). On the other hand, we wondered whether TCLP 1 minor accumulations at the posterior region of certain parasites (Figs 3 and 4) corresponded to late endosomal, “lysosome-like” compartments called reservosomes [12]. Cruzipain (CZP), the major *T*. *cruzi* cysteine-protease, constitutes a positive marker for these degradative compartments [39]. Co-staining experiments, however, indicated that TCLP 1 posterior accumulations were close to CZP-positive compartments, but no co-localization was evident (Fig 5). Finally, to analyze whether TCLP 1 had any association with protein ubiquitylation, another functional marker of active endocytosis of type I trans-membrane molecules in trypanosomatids [10], a mAb that stains for mono and poly-ubiquitylated proteins (FK-2) was used to probe wild type epimastigote and amastigote forms. As shown in Fig 5, FK-2 stained the cytoplasm, nucleus and kinetoplast of both developmental forms, which is consistent with the localization of the 20S proteasome in *T*. *cruzi* [54]. However, in addition to these compartments, FK-2 labeled a region close to the FP, where it indeed co-localized with TCLP 1 (Fig 5). To explore whether this co-localization was due to TCLP 1 proximity/interaction with ubiquitylated molecules and/or to the ubiquitylation of TCLP 1 itself at its UBL-Domain [15] we carried out immunoprecipitation assays. Unfortunately, and despite several attempts, we were not able to immunoprecipitate the TCLP 1::3xFLAG product from transgenic parasites (data not shown). Overall, these results indicate that TCLP 1 co-localizes with molecular and functional markers of the FP area.

### TCLP 1 over-expressing parasites show impaired survival at late stationary phase

**Fig 6:**
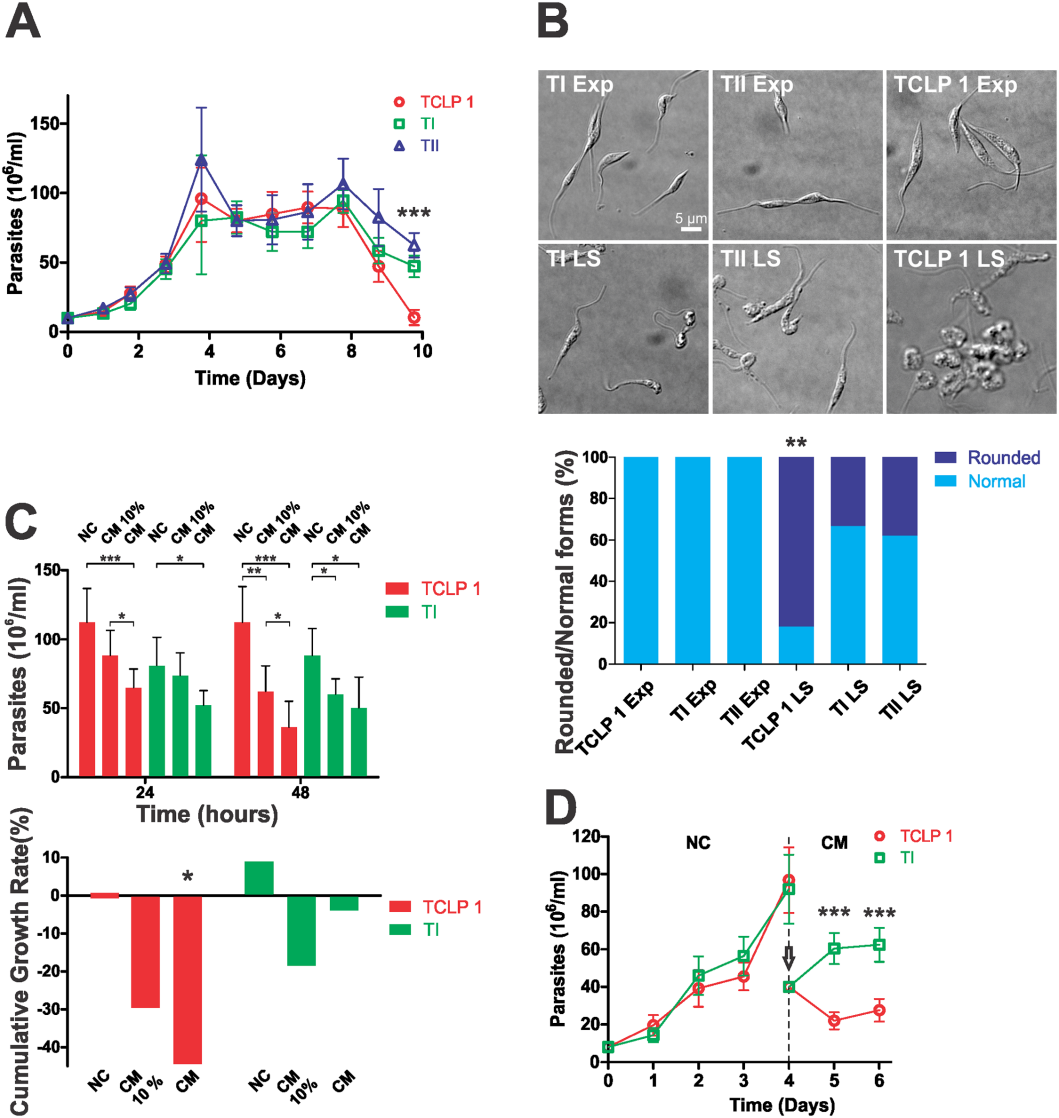
TCLP 1-transfected parasites show accelerated progression to late stationary phase of growth. (A) Adriana epimastigotes transfected with TCLP 1::3xFLAG (TCLP 1) or with TCLP 1-unrelated genes TcCLB.511277.250 (TI) or TcCLB.510181.60 (TII) were taken at the indicated time points, fixed, appropriately diluted and counted in a Neubauer chamber. Asterisks (***) denote significant differences (*p*<0.001) between TI or TII with respect to TCLP 1 as evaluated by Student’s T-test. (B) Above, Representative DIC images of TI, TII and TCLP 1 parasites in exponential (Exp) and Late Stationary (LS) growth phases are shown. Below, Parasites showing either normal or rounded morphology were counted for each parasite strain in each growing phase and the Rounded/Normal ratios (in%) are indicated. Asterisks (**) denote significant differences (*p<*0.05) between TCLP 1 LS and TI or TII during the LS phase. (C) Above, TCLP 1 and TI parasites were grown as in (B), but alternatively switched to NC (normal conditions, i.e. BHT 10% FCS), CM (conditioned, exhausted medium) or CM supplemented with 10% FCS (CM 10%). After 24 and 48 h, parasite numbers were determined for each strain growing under each condition. Asterisks (***, **, *) denote significant differences (*p<*0.001, *p<*0.01 and *p<*0.05, respectively) between parasites numbers as evaluated by Student’s T-test. Below, Cumulative Growth Rate values (in %) for the parasite density variation in each condition between 24 and 48 h. Asterisk (*) denotes significant differences (*p*<0.05) between TI and TCLP 1 as evaluated by Student’s T-test. (D) TCLP 1 and TI parasites were grown under NC until they reached the exponential phase of growth at day 4, when they were diluted to half (vertical arrow) and switched to CM for 2 additional days. Asterisks (***) denote significant differences (*p*<0.001) between TI and TCLP 1 as evaluated by Student’s T-test.

Standard growth curves in the absence of G418 indicated that TCLP 1 epimastigotes have altered growth kinetics as compared to wild type (S5 Fig). This phenotype has been previously observed for different *T*. *cruzi* transgenic lines, and is apparently a non-specific effect of transfection and/or growing the parasites under constant drug pressure [29, 55]. For comparison purposes, we generated in parallel 2 epimastigote strains (termed TI and TII) bearing 2 different *T*. *cruzi* genes having no *a priori* relationship with *TCLP 1*, cloned in pRIBOTEX (see Materials and Methods). All parasite strains were transfected at the same time and grown under identical conditions (i.e. in the presence of G418). As shown in Fig 6A, recombinant strains showed no significant growth differences at exponential (Exp, 0–4 days) and stationary (4–8 days) phases. TCLP 1 epimastigotes, however, showed a significant decay in their numbers at day 10 of late stationary phase (LS, 8–10 days), as compared to both TI and TII (Fig 6A). These differences were not correlated with an altered metacyclogenesis rate (not shown) but, instead, with a concomitant increase in the number of spheromastigotes (i.e. rounded, non-dividing and non-infective forms) at late stationary phase (Fig 6B).

In another experimental set-up, TI and TCLP 1 epimastigotes were allowed to grow until they reached the stationary phase, and then placed either in rich medium (normal conditions, NC), in conditioned medium (CM) or in CM supplemented with 10% fetal calf serum (CM 10%). The main aim of this experiment was to evaluate whether it was possible to restore the TCLP 1-determined growth phenotype by increasing nutrients availability in the CM. Parasites were counted at 24 and 48 h after switching to NC, CM or CM 10% (Fig 6C). Consistent with results shown in Fig 6A, TCLP 1 and TI epimastigotes did not display significant differences in cell density at NC in either time point. However, TCLP 1 epimastigotes showed a patent growth phenotype as compared to TI upon switching to CM, which was partially restored by the addition of 10% of fetal calf serum. Statistical analysis confirmed that TCLP 1 epimastigotes exhibited an overall augmented decay in CM conditions (Fig 6C and S3 Table). In order to better depict these differences, the Cumulative Growth Rate (CGR) for each strain growing under each condition was calculated and plotted as % value. As shown in Fig 6C, the only significant CGR value was found for TCLP 1 growing in the presence of CM (see S4 Table for significance values and complete statistical contrasts). Quite similar results were obtained when comparing TII and TCLP 1 epimastigotes (not shown).

It should be noted, however, that above assays were conducted at stationary or late stationary phases. Thus, we wanted to evaluate whether a similar growth phenotype could be evidenced on the exponential phase. We therefore mimicked LS phase conditions by diluting exponentially-growing strains in an exhausted, conditioned medium (CM). Under these conditions, cell density of TCLP 1 epimastigotes significantly diminished with respect to the initial inoculum, and to TI or TII lines (Fig 6D and not shown). Taken together, these results suggest that TCLP 1 over-expression leads to a constitutive intracellular transport defect in epimastigotes, which only translates into a growth phenotype upon their culture in an exhausted medium.

### TCLP 1 over-expressing parasites show diminished endocytic activity

**Fig 7:**
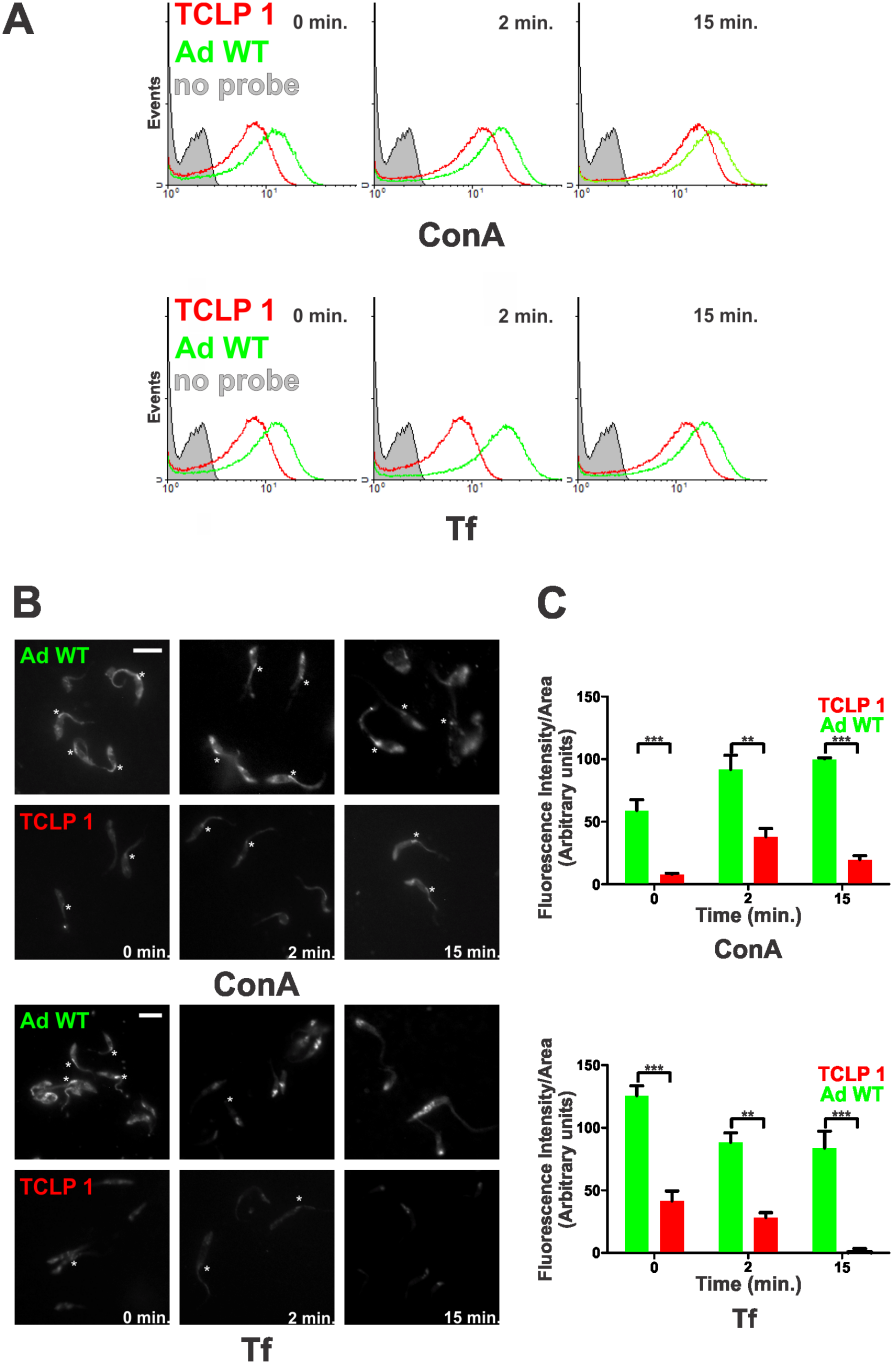
TCLP 1-transfected parasites show diminished endocytic capacity. (A) ConA-rhodamine (above) or Tf-Alexa 488 (below) internalization by either Adriana epimastigotes transfected with TCLP 1::3xFLAG (TCLP 1, red) or Adriana WT (Ad WT, green) parasites at the indicated time points was monitored by flow cytometry. Unmarked (no probe) parasites are shown in grey. (B) Representative epifluorescence images of Ad WT (upper panels) and TCLP 1 (lower panels) epimastigotes upon different times of incubation with ConA-rhodamine or Tf-Alexa 488 as indicated under Materials and Methods. C) Fluorescence quantification of dye labeling at the FP area (indicated with asterisks in (B)). Mean values of fluorescence (in Arbitrary Units) and standard deviation are plotted for TCLP 1 (dark grey) and Ad WT (light grey) parasites upon incubation with either dye. Asterisks denote significant differences (***, *p*<0.001; **, *p*<0.01) between Ad WT and TCLP 1 epimastigotes as evaluated by Student’s T-test.

Considering that i) TCLP 1 co-localized with endocytic markers at the FP area (Fig 5) and ii) its over-expression led to a growth phenotype, compatible with intracellular transport defect/s (Fig 6), we directly assessed the endocytic capacity of TCLP 1 epimastigotes. For these experiments, the dynamic uptake of the well established endocytic tracers ConA-rhodamine and Transferrin-Alexa 488 was followed in exponentially growing epimastigotes by flow cytometry [40] and by fluorescence quantitation at the FP region using microscopy techniques. Following a 10 min incubation period at 0°C, which allows binding of the tracers to the specific receptors at the FP membrane, parasites were placed at 28°C, a permissive temperature for membrane-bound endocytic processes. Aliquots were taken at 0, 2 and 15 min, extensively washed and analyzed by flow cytometry. For both tracers and for every time-point, our data indicated a generally diminished parasite-associated labeling for TCLP 1 epimastigotes as compared to the parental, wild type ones (Fig 7A). Interestingly, these differences were also evident at time 0, indicating that over-expression of TCLP 1 led to a decreased ConA and transferrin effective binding. As shown in Fig 7B, labeling for both tracers was observed both in the FP area and in posterior compartments of epimastigotes, in agreement with previously reported data [56]. Interestingly, both FP area and posterior compartments were generally more intensively labeled in wild type than in recombinant TCLP 1 parasites, thus in agreement with flow cytometry data (Fig 7A and 7B). Fluorescence quantitation of parasites populations confirmed this observation, by showing that binding of ConA-rhodamine and Transferrin-Alexa 488 to the FP was significantly lower in TCLP 1 parasites as compared to parental ones (Fig 7C). On the other hand, dye binding, uptake and increased between time points for both strains (. Thus, although additional downstream defects that may contribute to its reduced endocytic rate (i.e. at the trafficking to posterior degradative compartments and/or at the degradation of endocytic cargo proteins steps) cannot be ruled out, our results suggest that impaired (although not completely blocked) membrane-bound endocytosis in TCLP 1 parasites is due to a diminished number of steady-state FP surface receptors for macromolecules required for nutrition.

## Discussion

In this work, we identified and characterized a novel *T*. *cruzi* protein, termed TCLP 1, with structural homology to bacterial TIIISS chaperone CesT. According to our knowledge, this is the first report of this prokaryotic domain in an eukaryotic organism. We showed that TCLP 1 is a member of a family of chimeric proteins with a rather conserved architecture and restricted to trypanosomatids. We also showed that TCLP 1 expression is limited to *T*. *cruzi* replicative forms, where it localizes predominantly to the intracellular portion of the FP area, and could be associated with, or directly involved in, endocytic processes. Indeed, we demonstrated that TCLP 1 over-expression in epimastigotes leads to a reduced endocytic capacity and impaired survival under starvation conditions.

Based on our phylogeny analyses, the most parsimonious hypothesis suggests that the CEST domain was acquired by a trypanosomatid ancestor from the *Chlamydiaceae*. The idea of HGT from different Chlamydiae to the *Trypanosoma/Leishmania* group has been hypothesized before based on genome-wide comparisons [57]. Furthermore, one of such chlamydial proteins putatively transferred to trypanosomatids (NP_219546, mentioned by the authors as hypothetical) is indeed a CesT-like protein from *Chlamydia trachomatis* [57]. Importantly, and despite a very low sequence identity, the TCLP 1 CEST motif showed significant structural conservation with multi-cargo chaperones involved in the secretion of virulence factors through the TIIISS in enteropathogenic bacteria [17, 18]. Complementation assays using TCLP 1 or, more likely, solely its CEST motif should be conducted in order to determine whether the observed structural conservation has a functional correlate.

Two recently published reports suggested the existence of additional bacterial TIIISS elements into the genomes of trypanosomatids [58, 59]. In particular, an *in silico* phylogeny analysis indicated the conservation between *Salmonella* TIIISS effectors and the terminal regions of certain *T*. *cruzi* Mucin Associated Surface Proteins (MASPs) [59]. *MASP* genes code for highly polymorphic molecules, whose expression is up-regulated on infective trypomastigote forms and might be involved in the interaction with mammalian cells [60]. In the same line, it was reported that META1, a conserved *Leishmania* virulence factor display distant homology and structural similarity to *Shigella* MxiM, an outer membrane protein required for TIIISS-mediated effector translocation [58, 61]. Interestingly, META1 and other related molecules display several structural and functional features similar to TCLP 1 [62–65]. On one hand, their expression profiles are not homogeneous along the parasite life-cycle, suggesting they are under certain developmental regulation program. As previously shown [50], *TCLP 1* mRNA is up-regulated in replicative forms of *T*. *cruzi* (amastigotes and epimastigotes), which is consistent with our mRNA and protein expression data. Transcriptomic and proteomic data compiled in S2 Table indicate stage-specific expression for additional Class I and II "CEST-like" molecules in trypanosomatids [50, 63, 66]. Interestingly, META proteins were shown to be involved in *Leishmania* growth and infectivity [58, 64, 65]. Our present results show a somehow related sub-cellular localization and growth phenotype for TCLP 1. Moreover, genome-wide RNAi experiments in *T*. *brucei* indicate that down-regulation of the TCLP 1 paralog Tb.927.10.9240 also led to abnormal cell proliferation in blood-stream forms [67] (S2 Table). Overall, the existence of distinct molecules bearing sequence/structural conservation to TIIISS elements from bacterial origin and displaying quite similar structural and functional features in trypanosomatids generates many interesting evolutionary and mechanistic questions that deserve further exploration.

Microscopy-based and homologous expression system approaches support TCLP 1 accumulation at the *T*. *cruzi* FP area. So far, very little is known regarding FP sorting signals in trypanosomatids. *Cis*-acting motifs that direct cysteine-rich acidic trans-membrane protein (CRAM) and T-lymphocyte triggering factor (TLTF) to the FP area in *T*. *brucei* have been described [68, 69]. However, these sorting signals are highly divergent; while CRAM has a short N-terminal sequence, TLTF relies on an internal 144 amino acid-long region for its FP targeting. In the case of TCLP 1, accumulation at the FP may be achieved either directly by sequence and/or structural determinants, or indirectly via its interaction with carrier protein(s). In this latter sense, it is noteworthy that TCLP 1 bears a predicted PDZ domain, which has been shown to be involved in the targeting/scaffolding of multi-protein complexes in other systems [70]. Further assays such as TCLP 1 deletion mapping and co-immunoprecipitation should be carried out to address this issue.

Co-localization studies in *T*. *cruzi* parasites lines suggest that TCLP 1 is in close proximity (or associates to) membrane-related endocytic processes. In addition, TCLP 1 showed positive co-localization both with ConA and ubiquitin in the FP region. *In silico* analyses indicate that TCLP 1 bears an UBL-like domain, suggesting it may bind ubiquitin and/or ubiquitylated proteins [15]. Interestingly, ubiquitylation of proteins, among other roles, has been shown to be involved in proteasomal degradation of proteins, vesicle trafficking and regulation of endocytosis [71, 72]. Thus, we put forth the idea that TCLP 1 may be related to ubiquitin-dependent sorting during endocytosis in *T*. *cruzi* (see below). A similar process is known to be involved in trafficking and developmental expression regulation of surface proteins in African Trypanosomes [9, 10]. It is worth mentioning that the UBL domains present in “CEST-like” proteins are most similar to that found in Rad23, a protein that protects cytoplasmic proteins against proteasomal degradation [45, 73] and shuttles to the nucleus upon UV-induced DNA damage to assist in nucleotide excision repair activities [45]. Considering that TCLP 1 bears putative NLS/NES signals, and given its complex domain architecture, additional role(s) for this molecule in the parasite nucleus can be envisaged.

From a functional standpoint, our findings demonstrate that TCLP 1 over-expression leads to a significant decay in epimastigotes numbers at late stationary phases of axenic culture. As mentioned, we propose that this phenomenon is due (at least in part) to an endocytic defect, which is constitutively operating in transgenic epimastigotes but only translates into a growth phenotype upon their culture in an exhausted medium. The aggravated sensitivity to nutritional stress exhibited by TCLP 1 over-expressing parasites could be accounted for by a deficient uptake of nutrients or by an exacerbated accumulation of (or susceptibility to) harmful metabolic products and/or toxins present in the conditioned medium. Although not mutually exclusive, our findings showing that i) this growth phenotype can be partially restored by increasing nutrient availability in the conditioned medium and ii) TCLP 1 epimastigotes have a diminished endocytic capacity, better fit with the former hypothesis.

In *T*. *brucei* and higher eukaryotes, internalization and sorting of surface *trans*-membrane domain proteins require specific signals within the cytoplasmic domain that are recognized by multiple factors within the endocytic machinery. One such pathway involves covalent attachment of ubiquitin to substrate lysine residues within the cytoplasmic domain of trans-membrane proteins [9, 10]. Ubiquitylated endocytic cargo proteins are recognized by multiple proteins containing low-affinity ubiquitin-interacting motifs. Following endocytosis, ubiquitylated cargo is delivered to a specialized late endosome, the multivesicular body, mediated by the endocytic sorting complex required for transport, deubiquitylated and sorted for degradation. Considering that over-expression of TCLP 1 leads to a diminished binding of ConA and transferrin to the parasite FP, an active role of this molecule in the turnover and thus in regulating the number of surface receptors for macromolecules required for nutrition may be envisaged. Again, ubiquitin binding assays and functional analyses of TCLP 1 molecules lacking the UBL-like domain may help to clarify this issue.

As a final note, given their bacterial origin, putative function(s) and sub-cellular accumulation at the FP, a strategic place in the trypanosomatid body deeply involved in virulence, differentiation, survival and drug delivery [14], we propose TCLP 1 and related orthologs/paralogs as possible targets for intervention against these health-threatening parasites.

## Supporting Information

S1 Fig Alignment of CEST and "CEST-like" proteins. Complete protein alignment of TCLP 1 orthologs/paralogs in trypanosomatids and bacterial CEST proteins used to build the phylogenetic trees depicted in Fig 1. The TCLP 1 deduced product from the CL Brener gene (TcCLB.510241.10, upper sequence) is underlined, and its putative SP is indicated as a black shaded box. The annotated Methionine for each molecule, and Methionine 71 proposed by us to be the actual first residue in TCLP 1, are color-boxed. The UBL (dark blue box), CEST (red box), PDZ (grey box) domains and TCLP 1 peptide (black box) are shown. Abbreviations are defined as in legend to Fig 1.
(PDF)

S2 Fig Validation of TCLP I predicted ORF. A) The “P*-value* for coding” as function of nucleotide position for the TcCLB.510241.10 predicted ORF is shown. Predicted structural domains (SP,UBL,CEST,PDZ) are indicated above. Arrow and asterisk denote the position of initial Methionine (ATG codon), at position 211 bp. B) DNA sequence alignment between TcCLB.510241.10 predicted ORF and 2 representative clones of RT-PCR sequences obtained as indicated under Materials and Methods. SP cleavage site and splice-leader (SL) acceptor site are indicated by vertical arrows in the amino acid sequence and DNA sequence, respectively. Tc Me2 and TCLP 1 260 bp reverse primers used to amplify RT-derived products are denoted by arrows. C) RT-PCR products were separated by 1% agarose gel electrophoresis. First line (left): TCLP 1 PCR product obtained after the addition of reverse transcriptase (RT). Second line (middle): Negative control without RT. Last two lines: Molecular weight (bp) markers. D) Real-Time PCR analysis of *TCLP 1* transcript expression in *T*. *cruzi* developmental stages. Relative *TCLP I* mRNA abundance is shown for amastigotes (A), epimastigotes (E) and trypomastigotes (T) of *T*. *cruzi* CL Brener strain.
(TIF)

S3 Fig Validation of anti-TCLP 1 antibody. A) EGFP::TCLP 1- transfected Hela cells were probed either with rabbit anti-TCLP 1 affinity-purified antibody (αTCLP 1, upper panels) or with a non-related, affinity-purified rabbit serum (mock, lower panels). B) Total lysates of *E*. *coli* cells transformed with the *pTrcHis C* vector (pTrisC) or with a construct bearing the CEST motif in the same vector (pTrisC CEST) induced (ind) or not (unind) with IPTG were probed with an anti-6xHis antibody (αHis, upper panel) or αTCLP 1 (lower panel). Molecular markers (in kDa) are indicated to the left. C) Total lysates of Wild Type Adriana strain (Ad WT) and TCLP 1 epimastigotes were analyzed by Western blot using the anti-FLAG antibody (αFLAG, left panels) or αTCLP 1 (right panels). D) Ad WT (left) or TCLP 1 epimastigotes were analyzed by IIF using the αFLAG. E) Displacement assay of αTCLP 1 antibody in *T*. *cruzi* epimastigotes. CL Brener strain epimastigotes were probed with αTCLP 1 antibody, which was previously added with PBS (above panels), with 0.1 μg/μL of a non-related peptide (mock, middle panels) or with 0.1 μg/μL of the TCLP 1 peptide (bottom panels).
(TIF)

S4 Fig Analysis of TCLP 1 over-expression. Permeabilized, wild type Adriana epimastigotes (Ad WT, green) and Adriana epimastigotes transfected with TCLP 1::3xFLAG (TCLP 1, red) were labeled with the anti-TCLP 1 antibody (αTCLP 1) and analysed by flow cytometry. Isotype control (no Ab) is shown in grey.
(TIF)

S5 Fig TCLP 1 over-expression affects parasite growth. Wild type Adriana epimastigotes (Ad WT) and Adriana epimastigotes transfected with TCLP 1::3xFLAG (TCLP 1) were grown under standard conditions, without G418. Samples were taken at the indicated time points, fixed, appropriately diluted and counted in a Neubauer chamber. Asterisks (***) denote significant differences (*p*<0.001) between TCLP I and AdWT as evaluated by Student’s T-test.
(TIF)

S1 Table Primers used in this study. (DOC)

S2 Table Expression data and reported phenotypes of TCLP 1 and related molecules. (DOC)

S3 Table Statistical analysis of TCLP 1 growth curves (24 or 48 h). (DOC)

S4 Table Statistical analysis of TCLP 1 growth curves (24 vs. 48 h). (DOC)
