# Supplementary material for: A Novel Trypanosoma cruzi Protein Associated to the Flagellar Pocket of Replicative Stages and Involved in Parasite Growth
Source: PLoS One. 2015 Jun 18;10(6):e0130099. doi: 10.1371/journal.pone.0130099 (PMC4472858; doi:10.1371/journal.pone.0130099)
Supplement: S1 Fig — Complete protein alignment of TCLP 1 orthologs/paralogs in trypanosomatids and bacterial CEST proteins used to build the phylogenetic trees depicted in Fig 1. The TCLP 1 deduced product from the CL Brener gene (TcCLB.510241.10, upper sequence) is underlined, and its putative SP is indicated as a black shaded box. The annotated Methionine for each molecule, and Methionine 71 proposed by us to be the actual first residue in TCLP 1, are color-boxed. The UBL (dark blue box), CEST (red box), PDZ (grey box) domains and TCLP 1 peptide (black box) are shown. Abbreviations are defined as in legend to Fig 1. (PDF) [file pone.0130099.s001.pdf]

```
                             10        20        30        40        50        60
TCLP_I             MICYFLSLFFLNGEVAEVCFCTYVSFLFFFFFTSALFFSNNRKVERKLRGGKLLPGGWHT
MOQ_005905         ------------------------------------------------------------
TCSYLVIO_007169    ------------------------------------------------------------
LbrM.04.0910       ----------------------------------------------------------MS
LtaP04.0700        ----------------------------------------------------------MT
LmxM.04.0710       -----------------------------------------------------------M
LmjF.04.0710       -----------------------------------------------------------M
LinJ.04.0710       -----------------------------------------------------------M
LDBPK_040710       -----------------------------------------------------------M
TcCLB.504137.70    ------------------------------------------------------------
MOQ_003496         ------------------------------------------------------------
LbrM.31.0930       ------------------------------------------------------------
LmjF.31.0740       ------------------------------------------------------------
LmxM.30.0740       ------------------------------------------------------------
LinJ.31.0770       ------------------------------------------------------------
LDBPK_310770       ------------------------------------------------------------
TcIL3000.10.8010   ------------------------------------------------------------
Tb927.10.9240      ------------------------------------------------------------
Tbg972.10.11310    ------------------------------------------------------------
TvY486_1009120     ------------------------------------------------------------
TcCLB.504057.50    -----------------------------------------------------------M
MOQ_006328         ------------------------------------------------------------
TCSYLVIO_003797    ------------------------------------------------------------
LtaP34.3080        -----------------------------------MRVGVTVSVEPLFLSLYCTCCFFFW
LbrM.20.2750       ------------------------------------------------------------
LmxM.33.3170       ------------------------------------------------------------
LmjF.34.3170       ------------------------------------------------------------
LinJ.34.2950       ------------------------------------------------------------
LDBPK_342950       ------------------------------------------------------------
Chl.trachomatis    ------------------------------------------------------------
Simk.negevensis    ------------------------------------------------------------
Wad.chondrophila   ------------------------------------------------------------
Parachlamydia      ------------------------------------------------------------
Protochlamydia     ------------------------------------------------------------
Yersinia pestis    ------------------------------------------------------------
Salmonella(1jyoD)  ------------------------------------------------------------
Pseud. syringae    ------------------------------------------------------------
Salmonella(3epuA)  ------------------------------------------------------------
Escherichia coli   ------------------------------------------------------------
```

```
                         70        80        90       100       110       120
TCLP_I             LEPVPAKKDPMNANDVLTVISPDDGKRFRINIKGDIGRLTIGRLKQCLATASSYSVPVAD
MOQ_005905         ----------MNVNNVLTVISPEDGKRFRINIKGDIGRLTIGRLKQCLSTAS-YSVPVAD
TCSYLVIO_007169    ----------MNANDVLTVISPDDGKRFRINIRGDIGRLTIGRLKQCLAAASSYSVPIAD
LbrM.04.0910       TANAA-AAARSQPREHLNIIYPDEGKRYKLNVRAPISRLTVGKVKQCLAAVSSCPIPLNK
LtaP04.0700        SVNAVAAAAASQPREHLNIIYSDEGKRYKLSLRTPIARLTIGKVKQCLAAASSCPIPLRD
LmxM.04.0710       STVNAFPAAPPQPREHLNIIYSDEGKRYKLSLRTPIGRLTIGKVKQCLAAASSCPIPLQD
LmjF.04.0710       SAVDALAAAPSQPREHLNIIYADEGKRYKLSLRTPIGRLTIGKVKQCLAAASSCPIPLHD
LinJ.04.0710       SAVDALAAAPSQPREHLNIIYSDEGKRYKLSLRTPIGRLTIGKVKQCLAAASSCPIPLQD
LDBPK_040710       SAVDALAAAPSQPREHLNIIYSDEGKRYKLSLRTPIGRLTIGKVKQCLAAASSCPIPLQD
TcCLB.504137.70    -----------MDDKQAFFVQSEDGRKFKMVIRGDLGKLSVGKIRRYLKSYG-----IPD
MOQ_003496         -----------MDDKQAFFVQSEDGRKFKMVIRGDLGKLSVGKIRRYLKSYG-----IPD
LbrM.31.0930       -----------MEERQAFFVESTDGRKFKMVIRGDLGKLSVAKIRRYLRSYG-----VPE
LmjF.31.0740       ---------MQMEEKQAFFVESVDGRKFKMVIRGDLGKLSVAKIRRYLKSYG-----VPE
LmxM.30.0740       -----------MDEKQSFFVESSDGRKFKMVIRGDLGKLSVAKIRRYLKSYG-----VSE
LinJ.31.0770       -----------MEEKQAFFVESTDGRKFKMVIRGDLGKLSVAKIRRYLKSYG-----VPE
LDBPK_310770       -----------MEEKQAFFVESTDGRKFKMVIRGDLGKLSVAKIRRYLKSYG-----VPE
TcIL3000.10.8010   -----------MDDKQAFFVMGVDGRTFKMVIRGDLGKLSVGKIRRYLKSYG-----VQD
Tb927.10.9240      -----------MDDKQAFFVRCIDGKTFKMVIRGDLGKLTVGKIRRYLKSYG-----VQD
Tbg972.10.11310    -----------MDDKQAFFVRCIDGKTFKMVIRGDLGKLTVGKIRRYLKSYG-----VQD
TvY486_1009120     ---------MEADDKQAFYVQCADGRKFKMVIRGDLEKLSVGKIRRYLKSYG-----VKD
TcCLB.504057.50    TNKKKMSNSETGGTRCVYVLSDIDGFKYRLVMEGDVRLLTTSKLKRYLQNATGL---SAQ
MOQ_006328         -----MSKSETGGTRCVYVLSDIDGFKYRLVMEGDVRFLTTSKLKRYLQSATGL---SAQ
TCSYLVIO_003797    -----MSNSETGVIRCVYVLSDIDGFKYRLVMEGDVRLLTTSKLKRYLQNATGL---SAQ
LtaP34.3080        TVVMESSTTLATPTSCVYVLSDVDGYKYQLVMQGDVELLTVRKVKRYLQRAAGI---DPA
LbrM.20.2750       ---MELPSETAPPTTSVYVLSDIDGYKYKLVMQGDVELLSVRKVKRYLQRAAGI---HPA
LmxM.33.3170       ---MESSTAAAAPTTCVYVLSDVDGYKYKLVMQGDVQLLTVRKVKRYLQRAAGI---NPA
LmjF.34.3170       ---MESATAAAAPTACVYVLSDVDGYKYKLVMQGDLQLLTVRKVKRYLQRAAGI---DPA
LinJ.34.2950       ---MESSAAAAAPTTCVYVLSDVDGYKYKLVMQGDLQLLTVRKVKRYLQRAAGI---DPA
LDBPK_342950       ---MESSAAAAAPTTCVYVLSDVDGYKYKLVMQGDLQLLTVRKVKRYLQRAAGI---DPA
Chl. trachomatis   ------------------------------------------------------------
Simk. negevensis   ------------------------------------------------------------
Wad. chondrophila  ------------------------------------------------------------
Parachlamydia      ------------------------------------------------------------
Protochlamydia     ------------------------------------------------------------
Yersinia pestis    ------------------------------------------------------------
Salmonella(1jyoD)  ------------------------------------------------------------
Pseud. syringae    ------------------------------------------------------------
Salmonella(3epuA)  ------------------------------------------------------------
Escherichia coli   ------------------------------------------------------------
```

```
                                130       140       150       160       170       180
TCLP_I              QVIKFNGTPLTRDDEVCAAYGIMNGSTLTVEHRA--------------------------
MOQ_005905          QVIKFNGAPLTRDDEVCAAYGIMNGSTLTVEHRA--------------------------
TCSYLVIO_007169     QVIKFNGTPLTRDDEVCAAYGIMNGSTLTVEHRA--------------------------
LbrM.04.0910        MVLHLNGVPLSSDKDLCSSLGIGNGATLSLEARHTPNKYNSEEDGGDG-----------G
LtaP04.0700         MVLHLNGVPLSNDKDLCSTLGIDNGATLSVEPRHPSHQYNDDDGDGGRDAWQGHSPQMQG
LmxM.04.0710        MVLHLNGVPLSNDKDLCSSLGIGNGATLSVEPRHPPRKSNGGESDGDGDAWQRHSPRRRG
LmjF.04.0710        MVLHLNGVPLSNDKDLCASLGIGNGATLSVEPRHPPRRCNGGEGDGDGGAWQRHSPRMRG
LinJ.04.0710        MVLHLNGVPLSNDKDLCSSLGIGNGATLSVEPRHPPRQCNGGEGDGDGGAWQRHSPRMRE
LDBPK_040710        MVLHLNGVPLSNDKDLCSSLGIGNGATLSVEPRHPPRQCNGGEGDGDGGAWQRHSPRMRG
TcCLB.504137.70     GQLLLHDGVVLEDSAVGGDFGLKNNSMLQLVSPRRG------------------------
MOQ_003496          GQLLLYDGVVLEDSAVGGDFGLKNNSMLQLVSPRRGT-----------------------
LbrM.31.0930        NQQLLSGDRVLEDDMLGEDFGLEDEGVLHLWEPSVSR-----------------------
LmjF.31.0740        NQLLLAGNRVLEDAMLGEQFGLENEGVLHLREPTTVQ-----------------------
LmxM.30.0740        KQLLLAGNRVLEDTMLGEQFGLENEGVLQLRDPTTMQ-----------------------
LinJ.31.0770        NQLLLAGNRVLEDSMLGEQFGLENEGVLHLQEPTTMR-----------------------
LDBPK_310770        NQLLLAGNRVLEDSMLGEQFGLENEGVLHLQEPTTMR-----------------------
TcIL3000.10.8010    GMQLLFNGTALTDDQVGHEFGLCNNATLNLAP----------------------------
Tb927.10.9240       GMQLLFNGLSLADEQVGSDFGLCNGATLHLGPPSFSEPAVPQRTHGQ-------------
Tbg972.10.11310     GMQLLFNGLSLADEQVGSDFGLCNGATLHLGPPSFSETAVPQRTHGQ-------------
TvY486_1009120      DLRLIFNGMVLDDKELGADFGLHNNAVLQLEDPVPER-----------------------
TcCLB.504057.50     QQELSFRGRVMQDGECGGDVGLVDGAVLQLRHVKGMRRTNSGDGRR-----VNRLPSSSM
MOQ_006328          QQELSFRGRVMQDGECGGDVGLVDGAVLQLRHVKEMRRTHSGDGRC-----VNRLSSSSM
TCSYLVIO_003797     QQELSFRGRVMQDGECGGDVGLVDGAVLQLRHVKRMRRSNSGDGRRVNRLPSSSMSMGGA
LtaP34.3080         QQLLSFNSVALDDTMTGKDAGLFDGAILRLQQVSSSPAALATGISTDAAWPYHSTLGGPG
LbrM.20.2750        QQLLSFNSVELNDTMSGKDAGFFDGAILRLQQVSPSSAGRATCTSASGASPRLSAHKGPG
LmxM.33.3170        QQLLTFNSVALHDTMSGKDAGFFDGAILRLQQVSSSSAALAKCISACGASPFHSSHGVPG
LmjF.34.3170        QQLLTFNSVALDDTMSGKEAGFFDGAILRLQQVYSSSAALAQCISADGSSPFHSAHGGPG
LinJ.34.2950        QQLLTFNSVALDDTMSGKDAGFFDGAILRLQQVSSSSAALAQCISVDGASPFHRAHGGPG
LDBPK_342950        QQLLTFNSVALDDTMSGKDAGFFDGAILRLQQVSSSSAALAQCISVDGASPFHRAHGGPG
Chl. trachomatis    ------------------------------------------------------------
Simk. negevensis    ------------------------------------------------------------
Wad. chondrophila   ------------------------------------------------------------
Parachlamydia       ------------------------------------------------------------
Protochlamydia      ------------------------------------------------------------
Yersinia pestis     ------------------------------------------------------------
Salmonella(1jyoD)   ------------------------------------------------------------
Pseud. syringae     ------------------------------------------------------------
Salmonella(3epuA)   ------------------------------------------------------------
Escherichia coli    ------------------------------------------------------------
```

```
                       190       200       210       220       230       240
TCLP_I           ----------------------------------------------NVDPTTDHTASFVS
MOQ_005905       ----------------------------------------------NVDPTRDHTASFAS
TCSYLVIO_007169  ----------------------------------------------NVDPTTDHTASFVS
LbrM.04.0910     AKGAQSPV--------------------------------GAATPGAAASPLLSISSPMS
LtaP04.0700      VNGTQSPV--------------------------------CAAAAGPTTSCSPSSPFPMS
LmxM.04.0710     ARDAQSPV--------------------------------GAAAAGATAPSSPSSPLPMS
LmjF.04.0710     ARGTPSPV--------------------------------GAAAAGSPASSSLSPPFPMS
LinJ.04.0710     ARGTQSPV--------------------------------GAAVAGSPASSSLSSPFPMS
LDBPK_040710     ARGTQSPV--------------------------------GAAVAGSPASSSLSSPFPMS
TcCLB.504137.70  ----------------------------------------TVAAEVRSKPTSDPTPHVSG
MOQ_003496       -----------------------------------------VAAEVRSKPTSNPTPHVSG
LbrM.31.0930     ----------------------------------------------RPETAAIPSAPPPQ
LmjF.31.0740     ----------------------------------------------HLAPAAVPSTAQ--
LmxM.30.0740     ----------------------------------------------HLAPAAVPSTAPQQ
LinJ.31.0770     ----------------------------------------------PLAPAAVP------
LDBPK_310770     ----------------------------------------------PLAPAAVP------
TcIL3000.10.8010 -----------------------------------------------VESSVQPPNLPIK
Tb927.10.9240    ----------------------------------------GFSDEAAGSRTGDPTDTPHS
Tbg972.10.11310  ----------------------------------------GFSDEAAGSRTGDPTDTPHS
TvY486_1009120   -------------------------------------------------------AAEDS
TcCLB.504057.50  SMGGVAHR--------------------------------GRSSVVDAASCASPTAGLLT
MOQ_006328       SMGGAAHR--------------------------------GRSTVVDAASCVSPTAGLLT
TCSYLVIO_003797  AHR-------------------------------------GRSSVVDAASCASPTAGLLT
LtaP34.3080      TASSFPAPPLGAEHGGNRTSTGRKDFFLEETPSGMSATM-TIPALRAPQQMSNPRDLPAP
LbrM.20.2750     SASSNTV-SFRTQDNDSRTSPRKGDFVVDGTSALISAPAVRATIHTQQVVSSTPRALPPP
LmxM.33.3170     CASLLTAPPRGAQDPDTRTSPRRDGSFFAETSPRLSAPA-ARGVVGVPQLIGSPRALPPP
LmjF.34.3170     CASSFTAPPRGAQDPGTRTSPRGNGSFFAEASPRLSAPA-TRGAVGASQLISSPHALPPL
LinJ.34.2950     CSSSFTALPRGEQDTGTRTSPRGDGSFFAETSPRLSASA-ATGVVGAPQLISSPRALPPP
LDBPK_342950     CSSSFTALPRGEQDTGTRTSPRGDGSFFAETSPRLSASA-ATGVVGAPQLISSPRALPPP
Chl.trachomatis  ------------------------------------------------------------
Simk. negevensis ------------------------------------------------------------
Wad. chondrophila------------------------------------------------------------
Parachlamydia    ------------------------------------------------------------
Protochlamydia   ------------------------------------------------------------
Yersinia pestis  ------------------------------------------------------------
Salmonella(1jyoD)------------------------------------------------------------
Pseud. syringae  ------------------------------------------------------------
Salmonella(3epuA)------------------------------------------------------------
Escherichia coli ------------------------------------------------------------
```

```
                         250       260       270       280       290       300
TCLP_I            PHRQALEYQTIR-----QQEEILGDEVLGHDNTLQVKIDQLQDQVEAAEKKKMKIAREKE
MOQ_005905        PHRQALEYQTIR-----RQEEILSDEVLGQENTLQVKIDQLQDQVETAEKTRMRIEREKE
TCSYLVIO_007169   PHRQALEYQTIR-----RQEEILGDEVLGYDNTLQVKIDQLQDQVEAAEKKKMKIAREKE
LbrM.04.0910      HQRRLLELQTLQ-----QQDEMLHKDAVVRNDVLRHTLHHVEEEMDQANLRRRQLERQRE
LtaP04.0700       RQRRLLELQTLQ-----QQDEMLHKDSVVRDDVLQHTMHHVEEEMDRASVRRRQLQRQRE
LmxM.04.0710      RQRRLLELQTLQ-----QQDAMLHNDNVVRDDVLRHTMHRVEEEIDQANLRRRQLQRQRE
LmjF.04.0710      RQRRLLELQTLQ-----QADEMLRKDDAVRDDVLRHTMHHVEEEMDHANLHCRQLQRQRE
LinJ.04.0710      RQRRLLELQTLQ-----QQDEMLRKDDVVRDDVLRHTMRHVEEEMDQANLRRRQLQRQRE
LDBPK_040710      RQRRLLELQTLQ-----QQDEMLRKDDVVRDDVLRHTMRHVEEEMDQANLRRRQLQRQRE
TcCLB.504137.70   EKRSSSTHRATSADTRARHSERPSSRPLTTATVSHVDPSSMSDGLSHTNV----------
MOQ_003496        EKRSSSTHRATSTDTHARHSERPSSRPLTTATVSHVDPSSMSDGLSQTNV----------
LbrM.31.0930      QQQQRPYAP------------------------------------------------QET
LmjF.31.0740      -----------------SRTQLQQKPELSYEP-------------------------QSA
LmxM.30.0740      QLHIISSSPTAQ-----SRAQLHQKPQLSYGP-------------------------QSA
LinJ.31.0770      --------PTTQ-----SRAQLQQKPQLSYGP-------------------------QSA
LDBPK_310770      --------PTTH-----SRAQLEQKPQLSYGP-------------------------QSA
TcIL3000.10.8010  SRGRSDSSASLR-----RTDIERGRDREQGGTSPPPRNTNSREYQTKAQKTQDDMPDRMN
Tb927.10.9240     SLSGDSRERN-------SKADSTHRDPCGG---LSHADPARGTATYSGGRYNVDKGIPVT
Tbg972.10.11310   SLSGDSRERN-------SKADSTHRDPCGG---LSHADPARGTATYSGGRYNVDKGIPVT
TvY486_1009120    REVEVVQPSKQQ-----RKVAAQGKRTSLSDPHVESRSSA-----------------TRG
TcCLB.504057.50   PLQ-GSHPRDGQ-----NGGEAFTSPPP--------------------------------
MOQ_006328        PLQ-GSQSRDGQ-----NGGEAFTSPPP--------------------------------
TCSYLVIO_003797   PLQ-GSHPRDGQ-----NGGEAFTSPPP--------------------------------
LtaP34.3080       LQRLSASQLTGGAEPVMYKAASCMGDVVAGLDYASLPVPEGTTDATGSTVPGVS-GGNSS
LbrM.20.2750      LRCLSPNQSTGFAEREAHDAASCAKRDTVADIVYPSQAERRSACSATVGTLPTTSWGTSS
LmxM.33.3170      LRRLSPNQLTGGAEREVYDAASCPSRDVVAGMAYSSPLELTGASSATASTPQAIPGGTSS
LmjF.34.3170      LRCLSPNQLAGGAKREAHDAVSCTRRDVVAGMAYSSPPELAGTTGTTASTPQVIPGRTSS
LinJ.34.2950      LRCLSPNKLTGGAECEAYDAASCRRRGVVAGVAYSSPPELAGTTSVTVSTPQVIPGRASS
LDBPK_342950      LRCLSPNKLTGGAECEAYDAASCRRRGVVAGVAYSSPPELAGTTSVTVSTPQVIPGRASS
Chl. trachomatis  ------------------------------------------------------------
Simk. negevensis  ------------------------------------------------------------
Wad. chondrophila ------------------------------------------------------------
Parachlamydia     ------------------------------------------------------------
Protochlamydia    ------------------------------------------------------------
Yersinia pestis   ------------------------------------------------------------
Salmonella(1jyoD) ------------------------------------------------------------
Pseud. syringae   ------------------------------------------------------------
Salmonella(3epuA) ------------------------------------------------------------
Escherichia coli  ------------------------------------------------------------
```

```
                           310       320       330       340       350       360
TCLP I             KAERELARLRLQEEVAEKQKRRLTQLREREAQRAARRAADLAARREQLKAEEEATRAVAL
MOQ_005905         KAERELARLRLQEEVAEKQKRRLTQLREREAQRAARRAADLAARREQLKAEEEAARAVAL
TCSYLVIO_007169    NAERELAKLRQQEEFAEKQKRRLTQLREREAQRAARRAADLAARREQLKAEEEATRAVAL
LbrM.04.0910       SADEELQHIKGKEAEAARERERFAMLQAQEAARASERATQLVERREQLRAEQEAARAVAM
LtaP04.0700        SAEEELRRINEKEAETARERERLVTLQAQEAARSTERAAQLAERREQLRAEQEAARAVAM
LmxM.04.0710       SAEEELQRMSEKEAEAARERERLALLRAQEAARATERAAQLMERREQLRADQEAARAVAM
LmjF.04.0710       SAEEELHRMKEKEAEAARERERLAVLRAQEAARATERAAQLVERREQLRAEQEAARAVAM
LinJ.04.0710       SAEEELQRMNEKEAEAARERERLAVLRAQEAARATERAAKLVERREQLRAEQEAARAVAI
LDBPK_040710       SAEEELQRMNEKEAEAARERERLAVLRAQEAARATERAAKLVERREQLRAEQEAARAVAI
TcCLB.504137.70    VSSTALESNMNSKNNGKNLGSSLSATLEVENSRLRDEVDRLHQELSELRGRQKCGDGGVR
MOQ_003496         VSSTALGSNMNSKNNGKNIGSSLSATLEVENSRLRDEVDRLHQELSELRGRQKCGDGGVR
LbrM.31.0930       WRD---EPAARSTQEMEREIERLQRQVDAAVARNADLENQLAQAANSSADYAAHRGASEA
LmjF.31.0740       WRETRYEPAAHDARQMQREIERLQRQVEAAEARNADLEHQLEQVAESSAPYPPRGGAGGA
LmxM.30.0740       WRETQYEPTTHDARQMERVIEQLQRQVEAAEARNADLEHQLAQAAKSSGAYPPRGGAGSA
LinJ.31.0770       RRETQYEPATNDARQMEREIERLQRQVEAAEARNADLEHQLAQVAKPSAAYPPRGGADGA
LDBPK_310770       RRETQYEPATNDARQMEREIERLQRQVEAAEARNADLEHQLAQVAKPSAAYPPRGGADGA
TcIL3000.10.8010   HVDPERAVVSKPATRCSFSKGGISPVKTSNERSSTPEPATRSRRTQS--EELETQQLREE
Tb927.10.9240      ATSATKTNIDRIPSADLTSHSGFPVALEVENQQLREQVELLRREVADLRQKNKAPRATVL
Tbg972.10.11310    ATSATKTNIDRIPSADLTSHSGFPVALEVENQQLREQVELLRREVADLRQKNKAPRATVL
TvY486_1009120     PVAQEDTTVQSHHADPESSLAARSVTCNSSGLKSARLASVKSNDGSGFAGTGPVGSGSRT
TcCLB.504057.50    ------REMNYRSKRMGREAHALEIENARLEERLLEMERRLANASSDWRLQEEIEQLKGI
MOQ_006328         ------RDVNYRSKRMGREAHALEIENARLQERLLETERRLANASSDWRLLEEIEQLKGT
TCSYLVIO_003797    ------REMNYRSKRIGREPHALEIENARLEERLLEMERRLANASSDWRLQEEIEQLKGI
LtaP34.3080        AANTVSGDVHAYHRELEEKVAALSIDNVRLREQLQAVGRQAAESCTNWKHEEEVKRLKAA
LbrM.20.2750       AVNANWDGAHAYCQELEGKVAALSIDNVRLREQLQAAARQAAESCTDWKYEEEVKQLKAT
LmxM.33.3170       APNAHGDGVQAYCRELEEKVAALSIDNVRLREQLQVVARQAAEACPDWRHEEEVKRLKAA
LmjF.34.3170       ASSADGDGVHVYYRELEERVAALSLDNVRLREQLQAVARQAAEACTDGRHEEELKRLKAA
LinJ.34.2950       APNADGDGFHVYYRELEEKVAALSLDNVRLREQLQVVARQAAEACTDGRHEEEVKRLKAA
LDBPK_342950       APNADGDGFHVYYRELEEKVAALSLDNVRLREQLQVVARQAAEACTDGRHEEEVKRLKAA
Chl. trachomatis   ------------------------------------------------------------
Simk. negevensis   ----------------MYEREKIQTKFKEETSM---------------------------
Wad. chondrophila  ------------------------------------------------------------
Parachlamydia      ------------------------------------------------------------
Protochlamydia     ------------------------------------------------------------
Yersinia pestis    ------------------------------------------------------------
Salmonella(1jyoD)  ------------------------------------------------------------
Pseud. syringae    ------------------------------------------------------------
Salmonella(3epuA)  ------------------------------------------------------------
Escherichia coli   ------------------------------------------------------------
```

```
                        370       380       390       400       410       420
TCLP I                  QKLDNERLRASLEQERAEYEAEKERLAKEREEYERRAKEREVNIRKKEIEIEQQILAAER
MOQ_005905              QKLDNERRRASLEQERAVYEAEKERLAKEREEYEQRAKEREVNIRKKEIEIEQQILAAER
TCSYLVIO_007169         QKLDNERRRASLEQERAEYEAEKERLAKEREEYEQRAKEREVNIRKKEIEIEQQILAAER
LbrM.04.0910            LKLENEKKKALLAQQQAFFNAERERAKREQENFEATTKARELELRAREIDIEHLRLARIR
LtaP04.0700             LKLENEKKKTLLAQQQAFFDAERERAQLEQENFEATTKAQELALRAREIDIEHLRLARIR
LmxM.04.0710            LKLENEKKKALLAQQQAFFDAERERAQLEQEHFEATAKTRELELRAREIDIEHLRLARIR
LmjF.04.0710            LKLENEKKKALLAQQQAFFDAERERAQLEQENFEATTKARELELRAREIDIEHLRLARIR
LinJ.04.0710            LKLENEKKKALLAQQQAFFDAERERAQLEQENFEATTKARELELRAREIDIEHLRLARIR
LDBPK_040710            LKLENEKKKALLAQQQAFFDAERERAQLEQENFEATTKARELELRAREIDIEHLRLARIR
TcCLB.504137.70         T-----------------------------------------------------------
MOQ_003496              T-----------------------------------------------------------
LbrM.31.0930            RGMS--------------------------------------------------------
LmjF.31.0740            RRMS--------------------------------------------------------
LmxM.30.0740            LRMS--------------------------------------------------------
LinJ.31.0770            RRMS--------------------------------------------------------
LDBPK_310770            RRMS--------------------------------------------------------
TcIL3000.10.8010        VELLRQELRELKNERSASYSGVQNNIVIE-------------------------------
Tb927.10.9240           RQ----------------------------------------------------------
Tbg972.10.11310         RQ----------------------------------------------------------
TvY486_1009120          ISPTLRDENRQLRAEVEILRRELAELQEQQHSVGNTRQK---------------------
TcCLB.504057.50         IAQLLREKEAVGRSAEEKWRAKEEELVKELDLMREERRRLHREQTLFEEEQRALVRSLEE
MOQ_006328              IAQLLREKEAAERSAEENWRAKEEELVKELDLMREERRRLHREQTLFEEEQRAVVRSLEE
TCSYLVIO_003797         IAQLLREKEAAGRSAEEKWRAKEEELVKELDLMREERRRLHREQTVFEEEQRALVRSLEE
LtaP34.3080             VAMLQKSVNDAERAAADRWRVKEEELVKELDLLREERRRLQEDASAQEAKLQELVHSMEG
LbrM.20.2750            VAMAQQGARDAERAAAHRWRVKEEELVKELDLLREERRRFQEESTTQEAKLQGLMHSMEG
LmxM.33.3170            VALAQESVRDAERAAADRWRVKEEELVKELDLLREERRRFQEESTAQEAKLQELVHSMEG
LmjF.34.3170            VAVAQESVKDAERAAANRWRVKEEELVKELDLLREERRRFHEESTAQEAKLRELVHSMEG
LinJ.34.2950            VAMAQEGVKDAERAAADRWRVKEEELVKELDLLREERRRFHEESTAQEAKLQELVHSMEG
LDBPK_342950            VAMAQEGVKDAERAAADRWRVKEEELVKELDLLREERRRFHEESTAQEAKLQELVHSMEG
Chl. trachomatis        ------------------------------------------------------------
Simk. negevensis        ------------------------------------------------------------
Wad. chondrophila       ------------------------------------------------------------
Parachlamydia           ------------------------------------------------------------
Protochlamydia          ------------------------------------------------------------
Yersinia pestis         ------------------------------------------------------------
Salmonella(1jyoD)       ------------------------------------------------------------
Pseud. syringae         ------------------------------------------------------------
Salmonella(3epuA)       ------------------------------------------------------------
Escherichia coli        ------------------------------------------------------------
```

```
                        430       440       450       460       470       480
TCLP_I           DRRELELSRLVSHQNRLLYHERVGKPPPPELTHEGMRRGEWSGNDPFSLPVATPEDKNVA
MOQ_005905       DKKELELSRLVSHQNRLLYHERLGKPLPPELMHEGMKRGEWSGNDSFSLPRATPEDKNVA
TCSYLVIO_007169  DRRELELSRLVSHQNRLLYHERVGRPPPPELTHEGMRRGEWSGNDPFSLPVATPEDKNVA
LbrM.04.0910     EARALDMDRRLAVQQRLNYYAQLGIEAPGALQREAAAL------------------LLTE
LtaP04.0700      EARALDADRRLAVKQRLHYYAQLGVEAPRALQRQAAAL------------------LLTE
LmxM.04.0710     EARALDMDRRLAVKQRLHYYAQLGVEAPRALQREAAAL------------------FLTE
LmjF.04.0710     EARALDMDRRLAVKQRLHYYAQLGVEAPRALQREAAAL------------------LLTE
LinJ.04.0710     EARALDMDRRLAVKQRLHYYAQLGVEAPRALQREAAAL------------------LLTE
LDBPK_040710     EARALDMDRRLAVKQRLHYYAQLGVEAPRALQREAAAL------------------LLTE
TcCLB.504137.70  ------------------------------------------------------------
MOQ_003496       ------------------------------------------------------------
LbrM.31.0930     ------------------------------------------------------------
LmjF.31.0740     ------------------------------------------------------------
LmxM.30.0740     ------------------------------------------------------------
LinJ.31.0770     ------------------------------------------------------------
LDBPK_310770     ------------------------------------------------------------
TcIL3000.10.8010 ------------------------------------------------------------
Tb927.10.9240    ------------------------------------------------------------
Tbg972.10.11310  ------------------------------------------------------------
TvY486_1009120   ------------------------------------------------------------
TcCLB.504057.50  QVRSQQ------------------------------------------------------
MOQ_006328       QVRSQQ------------------------------------------------------
TCSYLVIO_003797  QVRSQQ------------------------------------------------------
LtaP34.3080      EIRGLKYE------------------------------------------------LHDK
LbrM.20.2750     EIRGLKYE------------------------------------------------LHDK
LmxM.33.3170     EIRGLKYE------------------------------------------------LHDK
LmjF.34.3170     EIRGLKYE------------------------------------------------LHDK
LinJ.34.2950     EIRGLKYE------------------------------------------------LHDK
LDBPK_342950     EIRGLKYE------------------------------------------------LHDK
Chl. trachomatis ------------------------------------------------------------
Simk. negevensis ------------------------------------------------------------
Wad. chondrophila------------------------------------------------------------
Parachlamydia    ------------------------------------------------------------
Protochlamydia   ------------------------------------------------------------
Yersinia pestis  ------------------------------------------------------------
Salmonella(1jyoD)------------------------------------------------------------
Pseud. syringae  ------------------------------------------------------------
Salmonella(3epuA)------------------------------------------------------------
Escherichia coli ------------------------------------------------------------
```

```
                                490       500       510       520       530       540
TCLP I                 WGKPQEINNNNPPNQQWQLNQKQSYRQSDARHPSGPIKSEENATANAQFEDTYQNRPRPP
MOQ_005905             WRKTQENNNNL-FNQQWKLNQKQSYGQSDARHPSGPIKTEGNVTANAHFEDSYQNRPRPS
TCSYLVIO_007169        WGKPQEINNNNPPNQQWQLNQKQSYRQSGAPHPSGPIKSEENATANAQFEDSYQNRPRPP
LbrM.04.0910           KGKSDGDATNEALVGESSHHWPGVQSHPPNSKVSATALKGHTGKSAASALDTITDGLVRS
LtaP04.0700            EGESDDDAGGEEAVGDRRNHWPGAPPNRPKSSRGVTGLKSATAKAVARNIDTVRNDLVRS
LmxM.04.0710           EGETDDDAEGEEPVGDGCRRWPSAQRHRPNSKMSVTGFKGHTGKAVASSLDTDNNDLVCS
LmjF.04.0710           EGKTDDDAEDEEPAGNGSSHWPSAQRHGPDSKMSVTGLNGHTRQSVARTLDTNNNDLVRS
LinJ.04.0710           EGEIDDNAEDEEPAGDGSSHWPSAQRHGLNSKMSVTGFKSHTRKSVASAQDTSNNDLVRS
LDBPK_040710           EGEIDDNAEDEVPAGDGSSHWPSAQRHGLNSKMSVTGFKSHTRKSVASAQDTSNNDLVRS
TcCLB.504137.70        ------------------------------------------------------------
MOQ_003496             ------------------------------------------------------------
LbrM.31.0930           ------------------------------------------------------------
LmjF.31.0740           ------------------------------------------------------------
LmxM.30.0740           ------------------------------------------------------------
LinJ.31.0770           ------------------------------------------------------------
LDBPK_310770           ------------------------------------------------------------
TcIL3000.10.8010       ------------------------------------------------------------
Tb927.10.9240          ------------------------------------------------------------
Tbg972.10.11310        ------------------------------------------------------------
TvY486_1009120         ------------------------------------------------------------
TcCLB.504057.50        ------------------------------------------------------------
MOQ_006328             ------------------------------------------------------------
TCSYLVIO_003797        ------------------------------------------------------------
LtaP34.3080            EEALQATRLSLAEIQKEKRPCRDLPCRGAHSFS-------------------------RD
LbrM.20.2750           DEALQTARLSLAELQRQTSSWPDVARCGSDSVS-------------------------GS
LmxM.33.3170           DEALQAARLSLAEVQSEKCSWTDVARRGVDICP-------------------------PG
LmjF.34.3170           DEALQAARLSLAELRSEKRSWTDAVRRGVDICP-------------------------RG
LinJ.34.2950           DEALQAARLSLAEAQSEKRSRTDVARRGVDICP-------------------------RG
LDBPK_342950           DEALQAARLSLAEAQSEKRSRTDVARRGVDICP-------------------------RG
Chl. trachomatis       ------------------------------------------------------------
Simk. negevensis       ------------------------------------------------------------
Wad. chondrophila      ------------------------------------------------------------
Parachlamydia          ------------------------------------------------------------
Protochlamydia         ------------------------------------------------------------
Yersinia pestis        ------------------------------------------------------------
Salmonella(1jyoD)      ------------------------------------------------------------
Pseud. syringae        ------------------------------------------------------------
Salmonella(3epuA)      ------------------------------------------------------------
Escherichia coli       ------------------------------------------------------------
```

```
                                 550       560       570       580       590       600
TCLP_I              RAAENVVKMMLPGRDNFALAHDGNNVYNARENAIDNLVCMGRDLGLENTLEFD-NNNTCV
MOQ_005905          KAAENEVKVMLPGSENFALAHGGDNVYNARENALENLVCMGRDLGLENTLEFD-NNNTCV
TCSYLVIO_007169     RAAENVVKMMLPGRDNFALAHGGNNVYNARENAIDNLVCMGRDLGLENTLEFD-NNNTCV
LbrM.04.0910        RGSSSPPLSHSAQRPAVVAAVPDGSFYDARENAEENLRRLGEDLGLRDGLQFD-ESDTCV
LtaP04.0700         RGPSSSPLSHADQSATVVTTVPDGGFYDARENAEENLRRLGDDLGLREKLQFD-DSNTCI
LmxM.04.0710        RGPSSSPLSHAGQGVAAVAIMPDGGFYDARENAEENLRRLGDDLGLRDRLQFD-DSNTCV
LmjF.04.0710        RGPSSSPLSHADQRVAGVATVPDGGFYDARENAEENLRRLGDDLGLGDGLQFD-DSNTCV
LinJ.04.0710        RGPSSSPLSHAGHSVAAVATVPDGGFYDARENAEENLRRLGDDLGLRDGLQFD-DSNTCV
LDBPK_040710        RGPSSSPLSHAGHSVAAVATVPDGGFYDARENAEENLRRLGDDLGLRDGLQFD-DSNTCV
TcCLB.504137.70     ----------------------GPVLVDLLQNAKSNLQELGEELGV--SLQFD-MNLTCV
MOQ_003496          ----------------------GPVLVDLLQNAKSNLQELSEELGV--SLQFD-MNLTCV
LbrM.31.0930        ----------------------RSIDVDGLQVAKESLNMLSQKLQV--SLYFD-KNLTCV
LmjF.31.0740        ----------------------NSSDLDGLQVAKQSLKILAQKLGV--SLQFD-KNLTCV
LmxM.30.0740        ----------------------ESSDLDGLQVAKESLKILAQKLGV--SLQFD-KNLTCV
LinJ.31.0770        ----------------------ESSDLDGLQVAKESLKILAQKLGV--SLQFD-KNLTCV
LDBPK_310770        ----------------------ESSDLDGLQVAKESLKILAQKLGV--SLQFD-KNLTCV
TcIL3000.10.8010    ---------------------------DVLQNAKANLRGLGEELGV--TLNFD-SNLTCV
Tb927.10.9240       ----------------------DLTSIEVLQSAKSNLQELAEELGV--PLQFD-TNFTCV
Tbg972.10.11310     ----------------------DLTSIEVLQSAKSNLQELAEELGV--PLQFD-MNFTCV
TvY486_1009120      ----------------------DPELMDILQNAKANLQELSEELGV--PLQFD-MNLTCA
TcCLB.504057.50     ------QRLLEQGTTITQQTRQRQPAMNVAEKVEKSLKQLSLELNI-PQLHLD-DNDTCV
MOQ_006328          ------KRLLEQGTMITQQTRQLQPTMNISERVERSLKQLSLELSL-PQLHLD-DNDTCV
TCSYLVIO_003797     ------QKLLEQGTTITQQTRQRQPAMNVAEKVEKSLKQLSLELNL-PQLHLD-DNDTCV
LtaP34.3080         ESLHTLELAESHHARIPLHQRQHGGGTSIDELAQTALEYLTQAFESAFRLELEPDNDTCV
LbrM.20.2750        RIDHPFAPAASPHARIPLHQRQHGGSSSIDGLAEAALECLAQTFESAAPLKLDPDNDTCV
LmxM.33.3170        EASHPFAPAGPRHTRIPQDQPQHGGSASIDELAETALTYLTQAFESAAPLELEPENDTCV
LmjF.34.3170        EASHPFAPAASRHARIPLQQRQHGCSANIDELAETAMEYLTQAFESATPLELEPENDTCV
LinJ.34.2950        EANHPFAPAASRRARIPLHQRQHGGSSSIDELAETALEYLTQAFESATPLVLEPENDTCV
LDBPK_342950        EANHPFAPAASRRARIPLHQRQHGGSSSIDELAETALEYLTQAFESATPLVLEPENDTCV
Chl. trachomatis    ---------------------------MSRQNAEENLKNFAKELKL-PDVAFD-QNNTCI
Simk. negevensis    ----------------------------SLENAKANLREFGKELNL-EGLAFD-ENHTCI
Wad. chondrophila---------------------------MSFENAKQNLAEFGKELGL-EGLEFD-ENNTCI
Parachlamydia       ---------------------------MSFENAKENLKEFGKELGL-EGLEFD-ENNTCI
Protochlamydia      ---------------------------MSFENAKENLKEFGKELGL-EGLEFD-ENNTCI
Yersinia pestis     -----------------------------MRTYSSLLEEFATELGL-EEIETN-ELGHGA
Salmonella(1jyoD)-----------------------------LQAHQDIIANIGEKLGL--PLTFD-DNNQCL
Pseud. syringae     ------------------------------MTIQDLLNALAIRLES-RPLSLD-ANHLCC
Salmonella(3epuA)----------------------------MYSRADRLLRQFSLKLNT-DSIVFD-ENRLCS
Escherichia coli    ----------------------------MSSRSELLLDRFAEKIGV-GSISFN-ENRLCS
```

```
                       610       620       630       640       650       660
TCLP_I              ISVDGQYNLIVTFDATTERL-YIYSTLMTNIP----HDPVLRLRVYEFLMEGALLGREMC
MOQ_005905          ISVDGQYNLIVTFDATTERL-YIYSTLMTNIP----NDPVLRLRVYEFLMEGALLGREMC
TCSYLVIO_007169     ISVDGQYNLIVTFDAATERL-YIYSTLMTNIP----HDPVLRLRVYEFLMEGALLGREMC
LbrM.04.0910        ISIDGEYTLLVMYDTVTERL-YLYSTLLASLPPVVQATTEGRLKLYEFLLEASLLGREMC
LtaP04.0700         ISIDGEYTLLVMYDAATERL-YLYSTLLAALPPVVQATAEGRLKLYEFLLEASLLGREMC
LmxM.04.0710        ISIDGEYTLLVMYDAATERL-YLYSTLLASLPPVVQATAEGRLKLYEFLLEASLLGREMC
LmjF.04.0710        ISIDGEYTLLVMYDAATERL-YLYSTLLASLPPVVQATAEGQLKLYEFLLEASLLGREMC
LinJ.04.0710        ISVDGEYTLLVMYDAATERL-YLYSTLLASLPPVVQATTEGRLKLYEFLLEASLLGREMC
LDBPK_040710        ISVDGEYTLLVMYDAATERL-YLYSTLLASLPPVVQATTEGRLKLYEFLLEASLLGREMC
TcCLB.504137.70     IGTDERHTVLVTFDHATERL-YVYSTLLTQLP----VDAAVRVKLYELLLEGSLLGREVC
MOQ_003496          IGTDERHTVLVTFDHATERL-YVYSTLLTQLP----VDAAVRVKLYELLLEGSLLGREVC
LbrM.31.0930        VGTDEDFSILLTYDPATERL-YIYSSLLTELP----ENLPLRMKLYELLLEGSLLGREVC
LmjF.31.0740        VGTDADFSILLTYDPATERL-YIYSSLLTELP----DHPPLRMKLYEVLLEGSLLGREVC
LmxM.30.0740        VGTDADFSILLTYDPATERL-YIYSSLLTELP----DHPPLRMKLYEVLLEGSLLGREVC
LinJ.31.0770        VGTDADFSLLLTYDPATERL-YIYSSLLTELP----DHPPLRMKLYEVLLEGSLLGREVC
LDBPK_310770        VGTDADFSLLLTYDPATERL-YIYSSLLTELP----DHPPLRMKLYEVLLEGSLLGREVC
TcIL3000.10.8010    VGNDESHTVLVTFDSVTERL-YIYATLLTQLP----LDPEVRAKLYELLLEGSLLSREVC
Tb927.10.9240       VGDDESHTVLVTFDCVTERL-YIYSTLLTQIP----HDGEVRAKLYELLLEGSLLSREVC
Tbg972.10.11310     VGDDESHTVLVTFDCVTERL-YIYSTLLTQIP----HDGEVRAKLYELLLEGSLLSREVC
TvY486_1009120      VGREESQTVLVTFDYHTERL-YVYATLLTELP----SNMEVRVKLYEVLLEGSLLGREVC
TcCLB.504057.50     LPLGDGTNILVTLDTITERL-FMYAVIANSLP----SNAGKRLELFEMLLEGALLGRDMA
MOQ_006328          LPLGDGTNILVTLDTITERL-FMYAVIANSLP----SNAGERLRLFEMLLEGALLGRDMA
TCSYLVIO_003797     LPLGDGTNILVTLDTVTERL-FMYAVIANSLP----SNAGKRLELFEMLLEGALLGRDMA
LtaP34.3080         LPIDNDLNLLVTLDRETERL-YMYVTLLNHLP----SPPAQRLQLYEMLLEGALLGKDMA
LbrM.20.2750        IPVANGLNVLVTLDRETERL-FLYVPLLNRLP----SSLVQRMQLYEMLLEGALLGKDMA
LmxM.33.3170        VSVAKDLNVLVTLDRETERL-YLYVTLLNHLP----SSAAQRMQLYEMLLEGALLGKDMA
LmjF.34.3170        VPVADDLNVLVTLDRETERL-YLYVTLLNHLP----SSPTQRMRLYEMLLEGALLGKDMA
LinJ.34.2950        VPVANDLKVLVTLDRETERL-YLYVTLLNHLP----SSPVQRMQLYEMLLEGALLGKDMA
LDBPK_342950        VPVANDLKVLVTLDRETERL-YLYVTLLNHLP----SSPVQRMQLYEMLLEGALLGKDMA
Chl. trachomatis    LFVDGEFSLHLTYEEHSDRL-YVYAPLLDGLP----DNPQRRLALYEKLLEGSMLGGQMA
Simk. negevensis    LGIDNTFSLHLTYEPNSDRL-YLYSPILDGLP----KDDPTKLKLYEALLEGSMLGGQMA
Wad. chondrophila   LGIDDEFSLHLTYEPNSKRL-YLYSPLLDGLP----RDDKTRLKLYETLLEGSMLGGQMA
Parachlamydia       LGIDDEFSLHLTYEPNSKRL-YLYSPLLDGLP----RDDKTKLRLYEKLLEMSMLGGQMA
Protochlamydia      LGIDDEFSLHLTYEPNSKRL-YLYSPLLDGLP----RDEKTKLRLYERLLEGSMLGGQMA
Yersinia pestis     VTIDKIWVVHLAPINEKELVAFMRAGILTGQS----------QLYDILRKN--LFSPLS
Salmonella(1jyoD)   LLLDSD--IFTSIEAKDDIW-LLNGMIIPLSP----VCGDSIWR--QIMVINGELAANNE
Pseud. syringae     LKVNE---LDMTLERIEKQN-TLFVYLCVGTL----STPASSTLLSDILAANLFHYGSSD
Salmonella(3epuA)   FIIDNRYRILLT-STNSEYI-MIYG--FCGKP------PDNNNLAFEFL-NANLWFAENN
Escherichia coli    FAIDEIYYISLS-DANDEYM-MIYG-VCGKFP---TDNPNFALE----ILNANLWFAENG
                                                                      ######
```

```
                            670       680       690       700       710       720
TCLP I              GGGVGASIKNDFILLSSSIYL-PTSLPTTLSTLAPQFLFSLNKWREKLGELLSTVDTQK-
MOQ_005905          GGGVGASIKNDFILLSTSIHL-PTSLPTTLSTLVPQFLLSLNKWREKLGELLSTVDLQK-
TCSYLVIO_007169     GGGVGASIKNDFILLSSSIYL-PTSLPTTLSTLAPQFLFSLNKWREKLGELLSTVDMQK-
LbrM.04.0910        GGGVGSSIRNDFILMSASLYM-PTSQPWSLRTLAPQFLHCLQHWRTKLTEFLQTLEAQ--
LtaP04.0700         GGGVGASLRNDFVLMSASLYM-PTSQPWSLRTLAPQFLHCLRHWRKKLTEFLQALEAQ--
LmxM.04.0710        GGGVGASLRNDFVLMSASLYM-PTSQPWSLRTLAPQFLHCLRHWRAKLTEFLQTLEAQ--
LmjF.04.0710        GGGVGASLRNDFVLMSASLYM-PTSQPWSLRTLAPQFLHCLRHWRTKLTEFLQTLEAQ--
LinJ.04.0710        GGGVGASLRNDFVLMSASLYM-PTSQPWSLRTLAPQFLHCLRHWRTKLTEFLQTLEAQ--
LDBPK_040710        GGGVGASLRNDFVLMSASLYM-PTSQPWSLRTLAPQFLHCLRHWRTKLTEFLQTLEAQ--
TcCLB.504137.70     GGGIGMSLQSGIVLLSTSIPL-RNCSTSALKDIMPSFVETLGRWRSLINELLD-------
MOQ_003496          GGGIGMSLQSGIVLLSTSIPL-RNCSTSALKDIMPSFVETLGRWRSLINELLD-------
LbrM.31.0930        GGGIGLSMQNSVVVLTTSLPM-RHCSAAALCETLPLFVETLLRWRSLVDELLR-------
LmjF.31.0740        GGGVGLSTQNNVVVLTTSLPV-RHCSPEALCEVMPLFVETLVRWRSLIDELVR-------
LmxM.30.0740        GGGVGLSTQNNVVVLTTSLPV-RHCGPEALCEVMPLFVETLVRWRSLIDELLR-------
LinJ.31.0770        GGGVGLSTQNNVVVLTTSLPV-RHCSPEALCEVMPLFVETLVRWRSLIDELLR-------
LDBPK_310770        GGGVGLSTQNNVVVLTTSLPV-RHCSPEALCEVMPLFVETLVRWRSLIDELLR-------
TcIL3000.10.8010    GGGIGISPQNGVVLLSTTIPL-RHCTSSTLRDIMPVFVETVQRWRLLINELLS-------
Tb927.10.9240       GGGIGISPQNGLVLLSTTIPL-RHCNSSALKDIMPVFVETVARWRSLINELLN-------
Tbg972.10.11310     GGGIGISPQNGLVLLSTTIPL-RHCNSSALKDIMPVFVETVARWRSLINELLN-------
TvY486_1009120      GGGIGLSLRDSVVLLSTTIPL-RCCTSSALKETMPIFIETLQRWRSLINELLS-------
TcCLB.504057.50     TGGVGVCLRNELIIMNVCVDI-VHADEYALASIARPFMESVQHWSEAVKKMMPEKKN---
MOQ_006328          TGGVGVCLRNNLIIMNVCVDI-VHADEYALARIARPFMDSVQHWSEAVQKVMPPKEN---
TCSYLVIO_003797     TGGVGVCLRNELIIMNVCVDI-VHADEYALASIARPFMESVQHWSEEVKQMMPQKKN---
LtaP34.3080         GGGVGVSLESNLVLMSISANL-RHGGPTALAATAAPFAKAAQAWKKRIDALLNTYSGL--
LbrM.20.2750        GGGVGVSLEANLVLMSVSANL-RHSAASALAVTATPFVEAAQALTTRIDALLSTRRVF--
LmxM.33.3170        GGGVGVSLESNLVLMSISVNL-RHSAASALADAAAPFVKAAQAWTKSIDVLLNT------
LmjF.34.3170        GGGVGVSPESNLVLMSVSANL-RHSGASALAATAAPFVIAAQAWTKSIDVLLNT------
LinJ.34.2950        GGSVGVSLESNLVLMSVSANL-RHSGASALAATAAPFVKAAQAWTKSIDVLLNN------
LDBPK_342950        GGSVGVSLESNLVLMSVSANL-RHSGASALAATAAPFVNAAQAWTKSIDVLLNS------
Chl. trachomatis    GGGVGVATKEQLILMHCVLDM-KYAETNLLKAFAQLFIETVVKWRTVCSDISAGREPTVD
Simk. negevensis    GGGIGVAVKEELILMHCVLEMGAGADTSSLRRFAPLFVESVEKWRDRAKRIMEGHDVQI-
Wad. chondrophilaGGGVGVAVKEELILMHCTIDM-EHAVSSALRAFAPLYVETVEKWRKICTDVSEGREVTPE
Parachlamydia       GGGVGVAVQEELILMHCTIDM-EHAVPSALRAFAPLYVETVEGLRKLCAEISEGRDTASK
Protochlamydia      GGGVGVAVKEELILMHCTLDM-EHAVSSALRAFAPLYVETVEKWRKICSEVSEGRDEGAN
Yersinia pestis     GVIRCALDKDDHWLLWSQLNI-NDTSGTQLASVLTSLVDKAVTLS--CEPTMKKEEDDH-
Salmonella(1jyoD)GTLAYIDAAETLLLIHAITDL---TNTYHIISQLESFVNQQEALKNILQEYAKV------
Pseud. syringae     GAAFGLDEKNNEVLLFQRFDP-LRIDEDHFVSACVQMIEVAKIWRT---KLLHGHSAPL-
Salmonella(3epuA)GPHLCYDNNSQSLLLALNFSL-NESSVEKLECEIEVVIRSMENLYHILQDKGITLDTDYT
Escherichia coli    GPYLCYESGAQSLLLALRFPL-DDATPEKLENEIEVVVKSMENLYLVLHNQGITLENEH-
                    ##############                             ###############
```

```
                                730       740       750       760       770       780
TCLP I            --MTSPSKNGHSTIEDTNELDSRHTPEEKAPRRIPSSTRSSRTQSAESGAGR--------
MOQ_005905        --MTTPSKNGHATVEGTSELESRQTPDEKVPRRIASSTRSSRTHSAESAAGR--------
TCSYLVIO_007169   --MTSPSKNGHSTIEDTNELDSRHTPEEKASRRIASSTRSSRTHSTESGAGR--------
LbrM.04.0910      --ESHLQGGGIDTVPPSPPAAAPQPPQQHGYFTASSSQSPSLAHSPYRPSSAAIRIVSPR
LtaP04.0700       --DGLSQGGGTGAGSSTPSVVTAQSSQLPAHTSAAYSLSPFMAHSAPRAGSAATSIASPL
LmxM.04.0710      --EGHAQSGGTGAGPATPPAAARQPPQHHVYTSSASSLSPSMTRSAPRADSAATSVASPQ
LmjF.04.0710      --EGCGQGGGTGAVPATPPAAARQSPQHHVYTSAASSLSPSMARSAPHAGSAATSIASPH
LinJ.04.0710      --EGHGQSGGTGAVPATPPAAARQPPQHHVYTSAASSLSPSMARSAPHAGSAATSIASPH
LDBPK_040710      --EGHGQSGGTGAVPATPPAAARQPPQHHVYTSAASSLSPSMARSAPHAGSAATSIASPH
TcCLB.504137.70   ------------------------------------------------------------
MOQ_003496        ------------------------------------------------------------
LbrM.31.0930      ------------------------------------------------------------
LmjF.31.0740      ------------------------------------------------------------
LmxM.30.0740      ------------------------------------------------------------
LinJ.31.0770      ------------------------------------------------------------
LDBPK_310770      ------------------------------------------------------------
TcIL3000.10.8010  ------------------------------------------------------------
Tb927.10.9240     ------------------------------------------------------------
Tbg972.10.11310   ------------------------------------------------------------
TvY486_1009120    ------------------------------------------------------------
TcCLB.504057.50   ------------------------------------------------------------
MOQ_006328        ------------------------------------------------------------
TCSYLVIO_003797   ------------------------------------------------------------
LtaP34.3080       ------------------------------------------------------------
LbrM.20.2750      ------------------------------------------------------------
LmxM.33.3170      ------------------------------------------------------------
LmjF.34.3170      ------------------------------------------------------------
LinJ.34.2950      ------------------------------------------------------------
LDBPK_342950      ------------------------------------------------------------
Chl. trachomatis  TMPQMPQGGGGGIQPPPAGIRA--------------------------------------
Simk. negevensis  --ERPQAGMQPGHRPGGDQPKPGDRF-IKI------------------------------
Wad. chondrophilaMTKAVPGSGSPGQRGDEPKGIKI-------------------------------------
Parachlamydia     PKESDMLPGGLSGKKGNQGFIKI-------------------------------------
Protochlamydia    ASKSLPPQITGGGKQGQGFIKI--------------------------------------
Yersinia pestis   --RPSSSHLLV-------------------------------------------------
Salmonella(1jyoD)------------------------------------------------------------
Pseud. syringae   --ASSTRLTKAGLMLTMAGTIR--------------------------------------
Salmonella(3epuA)HHHHHH------------------------------------------------------
Escherichia coli  --MKIEEISSSDNKHYYAGR----------------------------------------
```

```
                                  790       800       810       820       830       840
TCLP I              ----------------------------------------PVIGIEATDSVMINGVPSRH
MOQ_005905          ----------------------------------------PMIGIEATDSVMINGVPSRH
TCSYLVIO_007169     ----------------------------------------PVIGIEATDSVMINGVPSRH
LbrM.04.0910        PRCLATSPSPSQASSSAAVAVTPLAAPYTSPPNGSA---IPVLGLEVTGTVLINGVPTHY
LtaP04.0700         PKCFAASLSPLQASN------------------GSAKRIIPVLGLEVTGTVLVNGVPTHY
LmxM.04.0710        PKSCATSPSPLQAFNTAALAATPLAAPHTSSSNGTAKRVLPVLGLEVTGTVLVNGVPTHY
LmjF.04.0710        PKSSAASPSPLQASNSAAVAVTPLAAPHTSSFNGSAKRVIPVLGLEVTGTVLVNGVPTHY
LinJ.04.0710        PKSSAASPSPLQAPNTAVVAATPLAAPYTSSFGGSAKRVIPVLGLEVTGTVLVNGVPTHY
LDBPK_040710        PKSSAASPSPLQASNTAVVAATPLAAPYTSSFGGSAKRVIPVLGLEVTGTVLVNGVPTHY
TcCLB.504137.70     ------------------------------------------------------------
MOQ_003496          ------------------------------------------------------------
LbrM.31.0930        ------------------------------------------------------------
LmjF.31.0740        ------------------------------------------------------------
LmxM.30.0740        ------------------------------------------------------------
LinJ.31.0770        ------------------------------------------------------------
LDBPK_310770        ------------------------------------------------------------
TcIL3000.10.8010    ------------------------------------------------------------
Tb927.10.9240       ------------------------------------------------------------
Tbg972.10.11310     ------------------------------------------------------------
TvY486_1009120      ------------------------------------------------------------
TcCLB.504057.50     ------------------------------------------------------------
MOQ_006328          ------------------------------------------------------------
TCSYLVIO_003797     ------------------------------------------------------------
LtaP34.3080         ------------------------------------------------------------
LbrM.20.2750        ------------------------------------------------------------
LmxM.33.3170        ------------------------------------------------------------
LmjF.34.3170        ------------------------------------------------------------
LinJ.34.2950        ------------------------------------------------------------
LDBPK_342950        ------------------------------------------------------------
Chl. trachomatis    ------------------------------------------------------------
Simk. negevensis    ------------------------------------------------------------
Wad. chondrophila   ------------------------------------------------------------
Parachlamydia       ------------------------------------------------------------
Protochlamydia      ------------------------------------------------------------
Yersinia pestis     ------------------------------------------------------------
Salmonella(1jyoD)   ------------------------------------------------------------
Pseud. syringae     ------------------------------------------------------------
Salmonella(3epuA)   ------------------------------------------------------------
Escherichia coli    ------------------------------------------------------------
```

```
                                  850       860       870       880       890       900
TCLP_I             NGGVLVVNVGGPAALAGIKPHDIIKKIDYKRVTSLRQFQREV-SRLIVGKMVPVVVERSD
MOQ_005905         NGGVLVVNVGGPAALAGIKPHDIIKKINYKRVTSLRQFQREV-SRLIVGKMVPVLLERSD
TCSYLVIO_007169    NGGVLVVNVGGPAALAGIKPHDIIKKINYKRVTSLRQFQREV-SRLIVGKMVPIVVERSD
LbrM.04.0910       EDGVLVVNAAGPSVLAGVQPNDFIEELNGVRIHNVGDFRRVIEEQLAPGMLVPVRINRGG
LtaP04.0700        QDGVLVVNAAGPSVLAGVQPNDLIEELNGMRIHNVGDFRRVIEEQLAPGMLVPMRINRGG
LmxM.04.0710       QDGVLVVNAAGPSVLAGVQPNDFIEELNGARIHNVGDFRRVIEEQLTPGMLVPVRINRGG
LmjF.04.0710       QDGVLVVNAAGPSVLAGVQPNDLIEALNGTRVRNVGDFRRVIEEELTPGMIVPVRINRGG
LinJ.04.0710       QDGVLVVNAAGPSVLAGVQPNDLIEELNGTRVRNVGDFRRVIEEQLTPGMLVPVRINRGG
LDBPK_040710       QDGVLVVNAAGPSVLAGVQPNDLIEELNGTRVRNVGDFRRVIEEQLTPGMLVPVRINRGG
TcCLB.504137.70    ------------------------------------------------------------
MOQ_003496         ------------------------------------------------------------
LbrM.31.0930       ------------------------------------------------------------
LmjF.31.0740       ------------------------------------------------------------
LmxM.30.0740       ------------------------------------------------------------
LinJ.31.0770       ------------------------------------------------------------
LDBPK_310770       ------------------------------------------------------------
TcIL3000.10.8010   ------------------------------------------------------------
Tb927.10.9240      ------------------------------------------------------------
Tbg972.10.11310    ------------------------------------------------------------
TvY486_1009120     ------------------------------------------------------------
TcCLB.504057.50    ------------------------------------------------------------
MOQ_006328         ------------------------------------------------------------
TCSYLVIO_003797    ------------------------------------------------------------
LtaP34.3080        ------------------------------------------------------------
LbrM.20.2750       ------------------------------------------------------------
LmxM.33.3170       ------------------------------------------------------------
LmjF.34.3170       ------------------------------------------------------------
LinJ.34.2950       ------------------------------------------------------------
LDBPK_342950       ------------------------------------------------------------
Chl. trachomatis   ------------------------------------------------------------
Simk. negevensis   ------------------------------------------------------------
Wad. chondrophila  ------------------------------------------------------------
Parachlamydia      ------------------------------------------------------------
Protochlamydia     ------------------------------------------------------------
Yersinia pestis    ------------------------------------------------------------
Salmonella(1jyoD)  ------------------------------------------------------------
Pseud. syringae    ------------------------------------------------------------
Salmonella(3epuA)  ------------------------------------------------------------
Escherichia coli   ------------------------------------------------------------
```

```
                                   910
TCLP_I             VLHTIFIRVGSA---
MOQ_005905         VLHTIFIKVGSA---
TCSYLVIO_007169    VLHNIFIRVGSA---
LbrM.04.0910       VAMVVTVHVEAARPL
LtaP04.0700        VAMVVTVCVEAGQSL
LmxM.04.0710       VAMVVTVRVEAGRSL
LmjF.04.0710       VAMVVTVRVEAGRSL
LinJ.04.0710       VAMVVTVRVEAGRAL
LDBPK_040710       VAMVVTVRVEAGRAL
TcCLB.504137.70    ---------------
MOQ_003496         ---------------
LbrM.31.0930       ---------------
LmjF.31.0740       ---------------
LmxM.30.0740       ---------------
LinJ.31.0770       ---------------
LDBPK_310770       ---------------
TcIL3000.10.8010   ---------------
Tb927.10.9240      ---------------
Tbg972.10.11310    ---------------
TvY486_1009120     ---------------
TcCLB.504057.50    ---------------
MOQ_006328         ---------------
TCSYLVIO_003797    ---------------
LtaP34.3080        ---------------
LbrM.20.2750       ---------------
LmxM.33.3170       ---------------
LmjF.34.3170       ---------------
LinJ.34.2950       ---------------
LDBPK_342950       ---------------
Chl. trachomatis   ---------------
Simk. negevensis   ---------------
Wad. chondrophila  ---------------
Parachlamydia      ---------------
Protochlamydia     ---------------
Yersinia pestis    ---------------
Salmonella(1jyoD)  ---------------
Pseud. syringae    ---------------
Salmonella(3epuA)  ---------------
Escherichia coli   ---------------
```
