# Supplementary figures and images for: A Novel Trypanosoma cruzi Protein Associated to the Flagellar Pocket of Replicative Stages and Involved in Parasite Growth
Source: PLoS One. 2015 Jun 18;10(6):e0130099. doi: 10.1371/journal.pone.0130099 (PMC4472858; doi:10.1371/journal.pone.0130099)

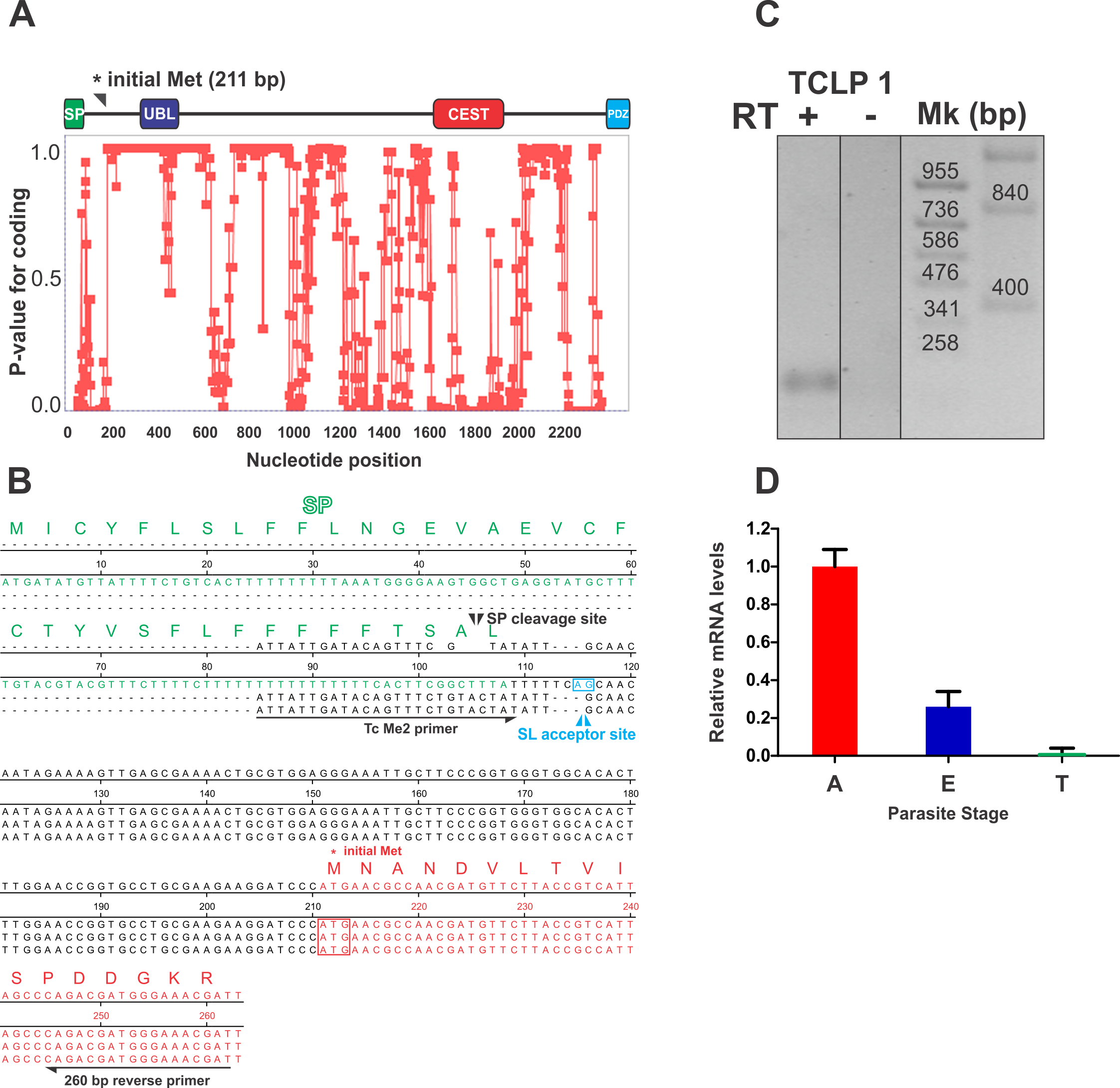

Supplement: S2 Fig — A) The “P-value for coding” as function of nucleotide position for the TcCLB.510241.10 predicted ORF is shown. Predicted structural domains (SP,UBL,CEST,PDZ) are indicated above. Arrow and asterisk denote the position of initial Methionine (ATG codon), at position 211 bp. B) DNA sequence alignment between TcCLB.510241.10 predicted ORF and 2 representative clones of RT-PCR sequences obtained as indicated under Materials and Methods. SP cleavage site and splice-leader (SL) acceptor site are indicated by vertical arrows in the amino acid sequence and DNA sequence, respectively. Tc Me2 and TCLP 1 260 bp reverse primers used to amplify RT-derived products are denoted by arrows. C) RT-PCR products were separated by 1% agarose gel electrophoresis. First line (left): TCLP 1 PCR product obtained after the addition of reverse transcriptase (RT). Second line (middle): Negative control without RT. Last two lines: Molecular weight (bp) markers. D) Real-Time PCR analysis of TCLP 1 transcript expression in T. cruzi developmental stages. Relative TCLP I mRNA abundance is shown for amastigotes (A), epimastigotes (E) and trypomastigotes (T) of T. cruzi CL Brener strain. (TIF) [file pone.0130099.s002.tif]

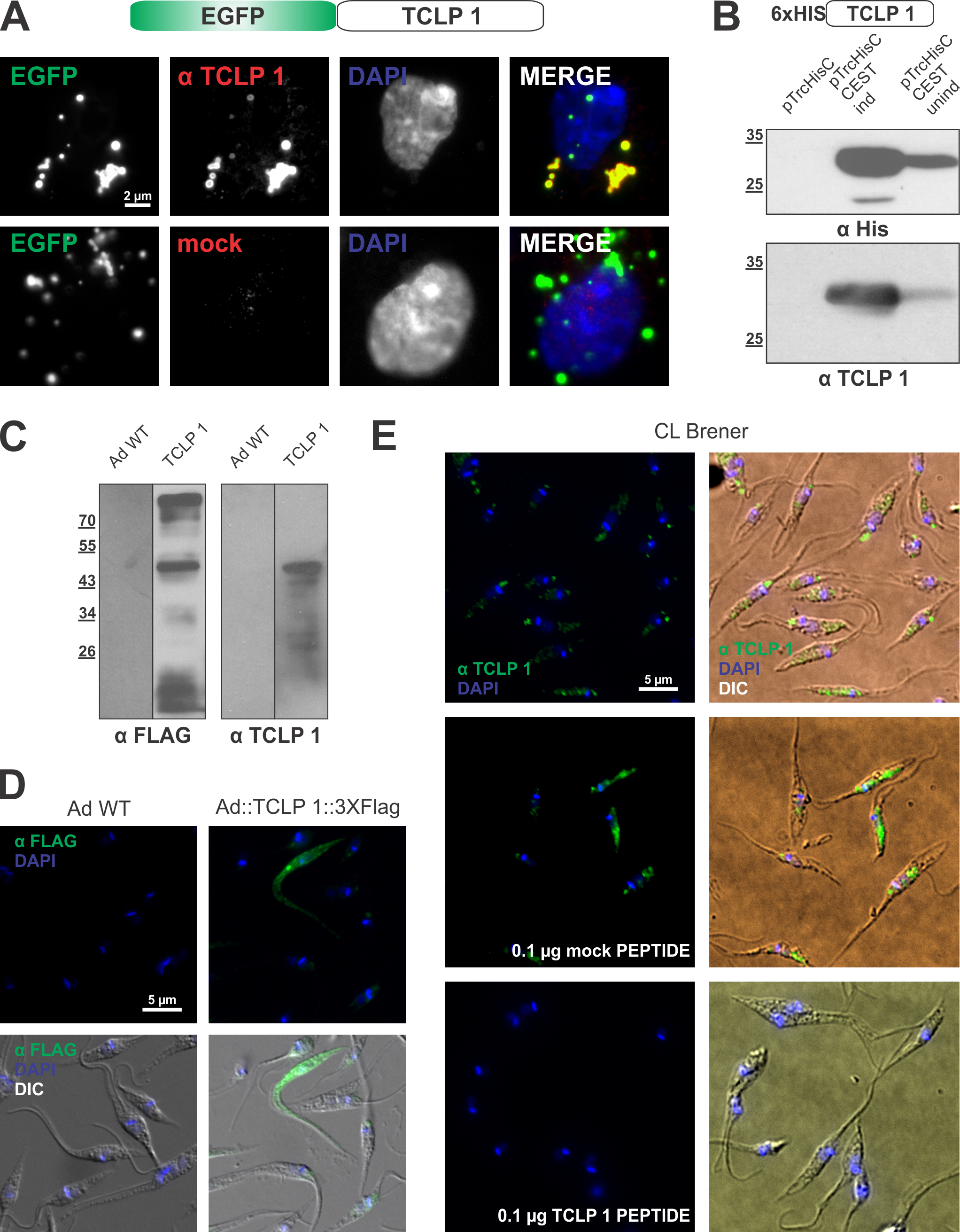

Supplement: S3 Fig — A) EGFP::TCLP 1- transfected Hela cells were probed either with rabbit anti-TCLP 1 affinity-purified antibody (αTCLP 1, upper panels) or with a non-related, affinity-purified rabbit serum (mock, lower panels). B) Total lysates of E. coli cells transformed with the pTrcHis C vector (pTrisC) or with a construct bearing the CEST motif in the same vector (pTrisC CEST) induced (ind) or not (unind) with IPTG were probed with an anti-6xHis antibody (αHis, upper panel) or αTCLP 1 (lower panel). Molecular markers (in kDa) are indicated to the left. C) Total lysates of Wild Type Adriana strain (Ad WT) and TCLP 1 epimastigotes were analyzed by Western blot using the anti-FLAG antibody (αFLAG, left panels) or αTCLP 1 (right panels). D) Ad WT (left) or TCLP 1 epimastigotes were analyzed by IIF using the αFLAG. E) Displacement assay of αTCLP 1 antibody in T. cruzi epimastigotes. CL Brener strain epimastigotes were probed with αTCLP 1 antibody, which was previously added with PBS (above panels), with 0.1 μg/μL of a non-related peptide (mock, middle panels) or with 0.1 μg/μL of the TCLP 1 peptide (bottom panels). (TIF) [file pone.0130099.s003.tif]

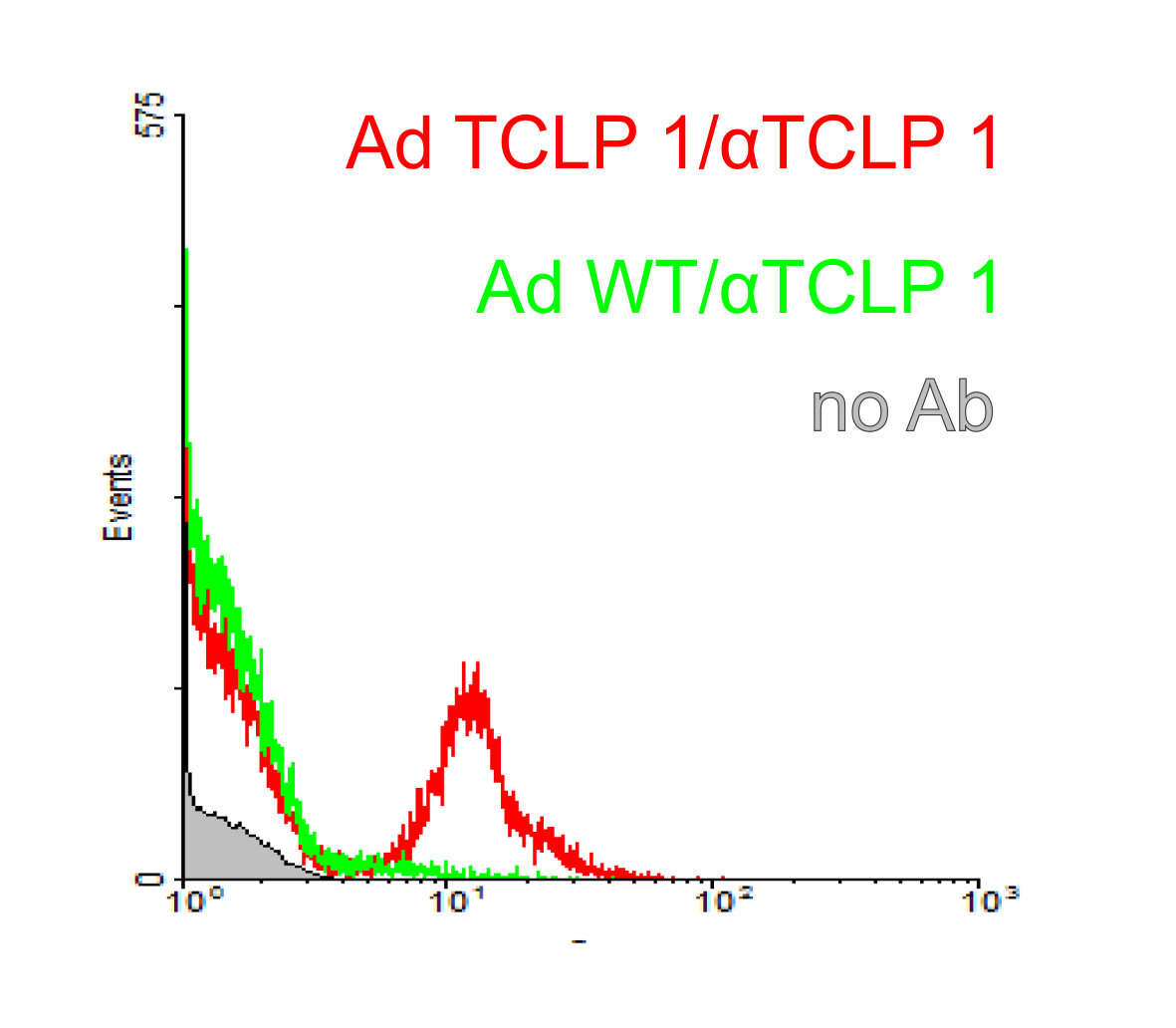

Supplement: S4 Fig — Permeabilized, wild type Adriana epimastigotes (Ad WT, green) and Adriana epimastigotes transfected with TCLP 1::3xFLAG (TCLP 1, red) were labeled with the anti-TCLP 1 antibody (αTCLP 1) and analysed by flow cytometry. Isotype control (no Ab) is shown in grey. (TIF) [file pone.0130099.s004.tif]

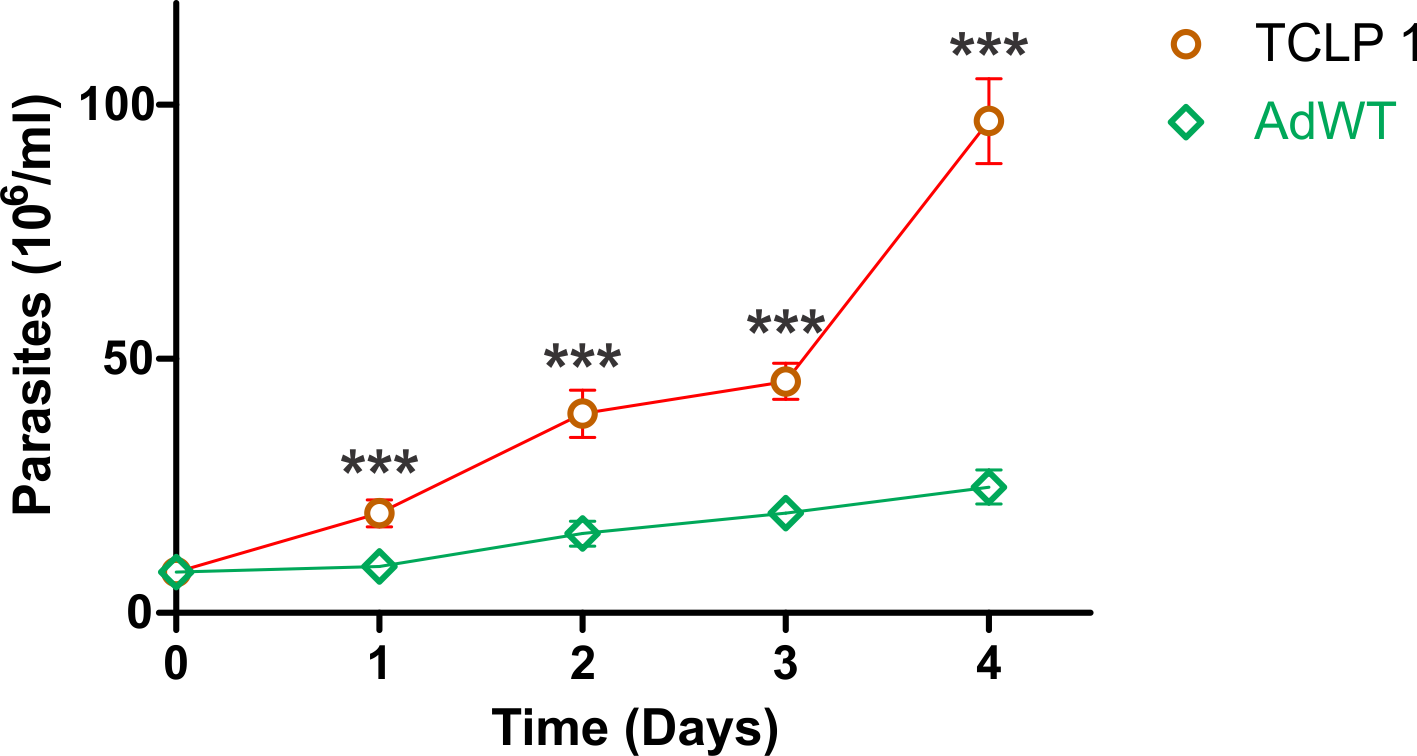

Supplement: S5 Fig — Wild type Adriana epimastigotes (Ad WT) and Adriana epimastigotes transfected with TCLP 1::3xFLAG (TCLP 1) were grown under standard conditions, without G418. Samples were taken at the indicated time points, fixed, appropriately diluted and counted in a Neubauer chamber. Asterisks (***) denote significant differences (p<0.001) between TCLP I and AdWT as evaluated by Student’s T-test. (TIF) [file pone.0130099.s005.tif]
