# Supplementary material for: A Novel Trypanosoma cruzi Protein Associated to the Flagellar Pocket of Replicative Stages and Involved in Parasite Growth
Source: PLoS One. 2015 Jun 18;10(6):e0130099. doi: 10.1371/journal.pone.0130099 (PMC4472858; doi:10.1371/journal.pone.0130099)
Supplement: S1 Table — (DOC) [file pone.0130099.s006.doc]

***Supplementary Table I.* Primers used in this study.**

| Primer name | Sequence (5’-3’)[[1]](#footnote-2) |
| --- | --- |
| TCLP 1 fwd | aaggatccatgaacgccaacga |
| TCLP 1 rev | tagcggccgcttgtcacacgcttgtaatct |
| CEST pTrcHis fwd | AAGAATTCATGTTGCCGGGCAGGGATAA |
| CEST pTrcHis rev | AAGAATTCTGCTTTTTCCTCTGGCGTGT |
| TcMe2 fwd | attattgatacagtttctgtactat |
| TCLP 1 260 bp rev | atcgtttcccatcgtct |
| rqF1 | TAGAACAAGAACGGGCAGAG |
| rqR1 | CCTTCTTGCGAATATTTACCTCAC |
| 18SF | ATACCTTCCTCAATCAAGAACC |
| 18SR | AAATAATCAAACCCGACCAC |

1. Restriction sites (when present) are underlined. [↑](#footnote-ref-2)
