# Supplementary material for: A Novel Trypanosoma cruzi Protein Associated to the Flagellar Pocket of Replicative Stages and Involved in Parasite Growth
Source: PLoS One. 2015 Jun 18;10(6):e0130099. doi: 10.1371/journal.pone.0130099 (PMC4472858; doi:10.1371/journal.pone.0130099)
Supplement: S2 Table — (DOC) [file pone.0130099.s007.doc]

***Supplementary Table II. Expression data and reported phenotypes of TCLP 1 and related molecules.***

| GI[[1]](#footnote-2) | Class | Expression evidence/Phenotype | Referenced |
| --- | --- | --- | --- |
| TcCLB.510241.10  (TCLP 1) | I | Epimastigote, Amastigote | [50], This work (TCLP 1) |
| TcCLB.504137.70 | II | Amastigote, Metacyclic |  |
| TcCLB.504057.50 | II | Epimastigote, Metacyclic |  |
| LinJ.04.0710 | I | Intracellular Amastigote | [62] |
| LinJ.31.0770 | II | Down-regulated in Amastigotes |  |
| LinJ.34.2950 |  |  |  |
| LmxM.04.0710 | I | Amastigote Secretome | [51] |
| LmxM.30.0740 | II |  |  |
| Tb.927.10.9240 | II | Abnormal growth, blood-stream form | [66] |
|  |  | Mild increase during heat-shock, procyclic form | [65] |

1. GI: Gene Identifier. [↑](#footnote-ref-2)
