# Supplementary material for: A Novel Trypanosoma cruzi Protein Associated to the Flagellar Pocket of Replicative Stages and Involved in Parasite Growth
Source: PLoS One. 2015 Jun 18;10(6):e0130099. doi: 10.1371/journal.pone.0130099 (PMC4472858; doi:10.1371/journal.pone.0130099)
Supplement: S3 Table — (DOC) [file pone.0130099.s008.doc]

***Supplementary Table III. Statistical analysis of TCLP 1 growth curves (24 or 48 hours).***

|  |  | **TCLP 1 NC** | **TCLP 1 CM 10%** | **TCLP 1 CM** | **TI NC** | **TI CM 10%** |
| --- | --- | --- | --- | --- | --- | --- |
| **24 hs.** | **TCLP 1 CM 10%** | 0.0939 (ns[[1]](#footnote-2)) |  |  |  |  |
|  | **TCLP 1 CM** | **0.0006 (***[[2]](#footnote-3))** | **0.0492 (*[[3]](#footnote-4))** |  |  |  |
|  | **TI NC** | 0.0586 (ns) | 0.6016 (ns) | 0.1756 (ns) |  |  |
|  | **TI CM 10 %** | **0.0012 (**[[4]](#footnote-5))** | 0.2251 (ns) | 0.3711 (ns) | 0.4950 (ns) |  |
|  | **TI CM** | **0.0005 (***)** | **0.0016 (**)** | 0.1520 (ns) | **0.0435 (*)** | 0.0623 (ns) |
| **48 hs.** | **TCLP 1 CM 10%** | **0.0046 (**)** |  |  |  |  |
|  | **TCLP 1 CM** | **<.0001 (***)** | **0.0497 (*)** |  |  |  |
|  | **TI NC** | 0.1500 (ns) | **0.0284 (*)** | **0.0005 (***)** |  |  |
|  | **TI CM 10 %** | **0.0027 (**)** | 0.8037 (ns) | **0.0486 (*)** | **0.0142 (*)** |  |
|  | **TI CM** | **0.0006 (***)** | 0.2088 (ns) | 0.3287 (ns) | **0.0118 (*)** | 0.3489 (ns) |

1. ns: non significant [↑](#footnote-ref-2)
2. ***: extremely significant [↑](#footnote-ref-3)
3. *: significant [↑](#footnote-ref-4)
4. **: highly significant

   Contingency tables showing p-values for each paired contrast (Student’s t-test) with a confidence level of 95% between TCLP 1 and Transfected I (TI) mean number of parasites at 24 (I), or 48 hours (II) after the switch to: Normal Conditions (NC), Conditioned Medium Supplemented with 10 % Fetal Calf Serum (CM 10%), or Conditioned Medium alone (CM). Differences are considered significant when p-values (bold, asterisks) are lower than 0.05. [↑](#footnote-ref-5)
