# Supplementary material for: A Novel Trypanosoma cruzi Protein Associated to the Flagellar Pocket of Replicative Stages and Involved in Parasite Growth
Source: PLoS One. 2015 Jun 18;10(6):e0130099. doi: 10.1371/journal.pone.0130099 (PMC4472858; doi:10.1371/journal.pone.0130099)
Supplement: S4 Table — (DOC) [file pone.0130099.s009.doc]

***Supplementary Table IV. Statistical analysis of TCLP 1 growth curves (24 vs. 48 hours).***

|  |  | **TCLP 1 48 hours** | | | **TI 48 hours** | | |
| --- | --- | --- | --- | --- | --- | --- | --- |
|  |  | **NC** | **CM 10%** | **CM** | **NC** | **CM 10%** | **CM** |
| **TCLP 1**  **24 hours** | **NC** | 1.0000 (ns) | **0.0051 (**)** | **<.0001 (***)** | 0.1269 (ns) | **0.0020 (**)** | **0.0018 (**)** |
|  | **CM 10%** | 0.1290 (ns) | 0.0703 (ns) | **0.0006 (***)** | 1.0000 (ns) | **0.0247 (*)** | **0.0261 (*)** |
|  | **CM** | **0.0040 (**)** | 0.8202 (ns) | **0.0308 (*)** | 0.0610 (ns) | 0.5539 (ns) | 0.2151 (ns) |
| **TI**  **24 hours** | **NC** | 0.0617 (ns) | 0.1606 (ns) | **0.0030 (**)** | 0.5805 (ns) | 0.0678 (ns) | 0.0642 (ns) |
|  | **CM 10 %** | **0.0064 (**)** | 0.3351 (ns) | **0.0006 (***)** | 0.1971 (ns) | 0.2146 (ns) | 0.1415 (ns) |
|  | **CM** | **0.0004(***)** | 0.2424 (ns) | 0.1799 (ns) | **0.0061 (**)** | 0.2775 (ns) | 0.8441 (ns) |

Contingency table showing p-values for each paired contrast (Student’s t-test) with a confidence level of 95% between TCLP 1 and TI mean number of parasites at 24 vs. 48 hours after the switch to: Normal Conditions (NC), Conditioned Medium Supplemented with 10 % Fetal Calf Serum (CM 10%), or Conditioned Medium alone (CM). Differences are considered significant when p-values (bold, asterisks) are lower than 0.05.
